# Beyond expectations: nocebo suggestion affects cognitive performance in older adults

**DOI:** 10.1007/s00426-026-02296-4

**Published:** 2026-05-15

**Authors:** Alessandra Barbon, Bernardo Villa-Sánchez, Mirta Fiorio, Sara Assecondi, Veronica Mazza

**Affiliations:** 1 Center for Mind/Brain Sciences (CIMeC), University of Trento, Rovereto, Italy; 2 Present Address: School of Biological and Health Systems Engineering, Arizona State University, Tempe, United States of America; 3 Department of Neurosciences, Biomedicine and Movement Sciences, University of Verona, Verona, Italy; 4 Department of Psychology and Cognitive Science (DiPSCo), University of Trento, Rovereto, Italy

**Keywords:** Nocebo effect, Placebo effect, Cognitive performance, Healthy aging, Expectation, Verbal suggestion, Online experiment

## Abstract

Placebo and nocebo effects are well-documented in pain perception and motor performance, yet their impact on cognitive performance remains unclear. Understanding these effects is relevant for designing interventions that leverage positive expectations to enhance cognitive outcomes while mitigating negative expectations, especially for older adults facing age-related cognitive decline. Furthermore, it is unclear whether these effects can be induced remotely, in the absence of direct participant-experimenter interactions. This study examined whether verbal suggestions about an intervention potential to enhance or impair cognitive performance could induce placebo and nocebo effects—both objective and subjective—in healthy older adults (aged 65–80) in an online setting. Participants completed an oddball task in auditory and visual modalities before (PRE) and after (POST) exposure to an inert acoustic intervention (i.e., the placebo/nocebo intervention). Prior to the intervention, individuals were randomly assigned to one of three groups, receiving neutral (Control), positive (Placebo), or negative (Nocebo) information about the intervention. The Nocebo group showed impaired performance at POST, consistent with the verbal suggestion, despite no negative shift in expectancy or perceived efficacy. In contrast, the Placebo group expected improvements and perceived benefits, but their performance did not differ from controls. This indicates a dissociation in objective and subjective components between placebo/nocebo effects in a perceptual and attentional task. Altogether, even without in-person interaction, nocebo effects are observable in cognitive performance in older adults. In contrast, placebo effects are limited to subjective beliefs, emphasizing the importance of additional factors such as motivation, and individual differences, in online contexts.

**Supplementary Information:**

The online version contains supplementary material available at 10.1007/s00426-026-02296-4.

## Introduction

Cognitive aging refers to the gradual changes in cognitive functioning that unfold across the lifespan as part of the natural aging process (Harada et al., 2013). With advancing age, certain cognitive abilities—such as processing speed, working memory, and executive functions—tend to decline, although not all domains are equally affected (Harada et al., 2013). These age-related cognitive changes can vary significantly between individuals and widely impact daily functioning and overall quality of life (Han et al., 2016; Harada et al., 2013). As a result, there has been a growing interest in identifying strategies to promote healthy cognitive aging, in particular those that can help preserve cognitive functioning and foster resilience in later life.

Among the numerous methods proposed to preserve cognitive function, one promising, yet underexplored, approach involves the manipulation of expectations. This line of inquiry builds on what are commonly known as *expectancy effects*, which are primarily driven by individuals’ belief that an intervention will influence their performance (Stewart-Williams & Podd, 2004). These effects can manifest in the form of placebo or nocebo effects, where beneficial or detrimental changes in outcomes arise from psychological mechanisms such as expectations, as well as contextual, verbal, and belief factors rather than the active components of an intervention (Benedetti et al., 2011; Crow et al., 1999; Hahn, 1997). Traditionally, such effects were considered as confounding variables to be minimized to isolate the specific effects of an active intervention. From this perspective, improvements driven by positive expectations were considered threats to internal validity. However, recent research challenges this view, suggesting that these effects can serve as active modulators of intervention outcomes (Colloca et al., 2023; Shawn Green et al., 2019).

Despite this shift in perspective, most of the research exploiting the placebo and nocebo effects has been more prominent in domains such as pain management, clinical studies and also in motor performance. In these areas, both behavioral and neurophysiological changes have been documented (for a recent review on placebo and nocebo effects in pain, see Colloca, 2019; for an overview in the motor domain, see Fiorio, 2018). In contrast, the investigation of these effects in the domain of cognitive performance—particularly among older adults—remains limited. To begin addressing this gap, a key approach is to experimentally influence expectations while keeping the same intervention but without its active component. This allows the specific contribution of expectancy effects to cognitive performance to be isolated (Joessel et al., 2025). However, the existing literature regarding placebo and nocebo effects on cognition has largely concentrated on younger adults; in addition, findings in this population have been mixed (Blokland, 2023; Colagiuri & Boakes, 2010; Looby & Earleywine, 2011; Schwarz & Büchel, 2015; Turi et al., 2017, 2018; Winkler & Hermann, 2019). Some studies have reported effects both at the subjective level (i.e., changes in the self-reported expectations or perceived outcomes) and at the objective level (i.e., changes in performance such as reaction times or accuracy) (Colagiuri & Boakes, 2010; Turi et al., 2018). Others have found changes only at the subjective level (Schwarz & Büchel, 2015; Winkler & Hermann, 2019) or only at the objective level (Turi et al., 2017), while some studies have reported no significant effects (Looby & Earleywine, 2011). In older adults, research is more limited, with only one study available to date (Oken et al., 2008). In this study, participants aged 65–85 taking a placebo pill described as a cognitive enhancer for two weeks showed improvements in delayed recall and choice reaction time. However, the effects were not consistent across all the cognitive domains assessed, and later analyses raised concerns about the robustness of the findings (Naqvi et al., 2013). Given that older adults may hold different beliefs about their cognitive abilities and expectations regarding their capacity for cognitive improvement compared to younger populations (Rabipour et al., 2018), further research specifically targeting this age group is needed.

An additional open question concerns whether placebo and nocebo effects can be effectively elicited in online environments. While most existing research has been conducted in face-to-face contexts, where participant–experimenter or patient-clinician interactions may influence the outcomes (see Howe et al., 2017), these settings may also increase the likelihood of demand characteristics or socially driven response biases. It is uncertain whether expectancy effects can occur remotely, where interpersonal dynamics and potential demand effects are minimized. Understanding whether verbal suggestions intended to explicitly manipulate expectancy remain effective in the absence of a direct personal interaction is relevant for expanding their applicability in remote interventions, such as online cognitive training programs and e-health applications. If placebo and nocebo effects on cognitive performance can emerge in online settings through the manipulation of expectations, they could be strategically leveraged to amplify beneficial responses and reduce detrimental ones, thereby enhancing the efficacy of these interventions; this, in turn, would increase inclusion and accessibility for individuals who may face barriers to in-person participation, such as older adults (Henrich et al., 2010; Woods et al., 2013).

The current study aims to address the issues described above by examining expectancy-driven placebo and nocebo effects on cognitive performance in older individuals, within an online setting. To this end, participants completed an oddball task, commonly used to assess attention, before and after being exposed to an inert intervention consisting of an acoustic frequency described as having cognitive enhancing properties (Placebo group) or detrimental effects (Nocebo group) on attention. The control group received neutral instructions, designed to elicit no specific expectations.

We first aimed to examine whether verbal suggestions of cognitive enhancement or impairment would influence behavioral performance and subjective beliefs (i.e., performance expectations and perceived intervention efficacy), thereby eliciting a placebo or nocebo effect. We hypothesized that positive suggestions would enhance objective performance and lead participants to expect and perceive improvements (objective and subjective placebo effects), whereas negative suggestions would impair objective performance and lead participants to expect and perceive worsening (objective and subjective nocebo effects). Second, we examined whether placebo and nocebo effects varied as a function of sensory modality (auditory vs. visual). Given the limited evidence on modality specificity in cognitive placebo/nocebo research, we explored whether such effects would generalize across modalities or emerge in a modality-dependent manner. Because the intervention consisted of an acoustic frequency, the auditory oddball task represented a unimodal condition (same modality as the intervention), whereas the visual oddball task represented a crossmodal condition (different modality).

## Methods

### Participants

All participants were recruited online through Prolific (www.prolific.com). Inclusion criteria were: age range between 65 and 80 years and an approval rate of over 90% on previous Prolific studies.

Participants were excluded according to the following criteria:

- Prescreening (self-reported): (1) reporting hearing loss or hearing difficulties; (2) not having normal or corrected-to-normal vision; (3) not being fluent in English; (4) having a diagnosed mild cognitive impairment (MCI) or dementia; and (5) having an ongoing or past diagnosis of a mental health illness or condition;
- Performance-based: (1) obtaining a score ≤ 26 on SATURN (Bissig et al., 2020; Tagliabue et al., 2023), indicating potential cognitive impairment; and (2) failing both headphone-use screening tests (Woods et al., 2017; Milne et al., 2021).

The estimated sample size was calculated using G*Power software (Version 3.1.9.4), considering a repeated measures analysis of variance with 3 groups (Placebo, Nocebo, Control) and 2 time points (PRE- and POST-intervention). In the absence of literature data to estimate the effect size of interest, we assumed a medium effect size (f) of 0.25 (Cohen, 2013), an alpha level of 0.05, a power (1-β) of 0.95, a correlation between repeated measures of 0.5, and a non-sphericity correction of 1. The resulting total sample size is 66 participants, with 22 participants per experimental condition.

A total of 204 older adults were initially recruited. Detailed information regarding participant flow, exclusions, and final sample sizes for each task is reported in the Results section.

### Materials, tasks and procedure

This study was conducted online and required participants to use a device other than a tablet or smartphone. To ensure proper sound delivery, participants were advised against using Firefox due to issues with audio file downloads, and the use of headphones was mandatory. There were two sessions, separated by an average interval of 3.33 days (SD = 2.15; range: 1–10 days). Each session involved the execution of different tasks, which were developed using Psychopy (v2023.2.23; Peirce et al., 2019), with Python and JavaScript code, and were hosted through Pavlovia (Pavlovia.org, Pavlovia Surveys; Open Science Tools, Nottingham, UK).

#### Session 1 – recruitment and screening phase

After providing written informed consent, participants answered demographic and screening questions. Informed consent, demographic information and exclusion criteria questions were all provided on Psytoolkit (psytoolkit.org; Stoet, 2010, 2017). Participants who met the initial eligibility criteria were asked to complete SATURN to assess cognitive ability, followed by two online headphone-use screening tests. This session lasted approximately 60 minutes. Participants’ scores were assessed only after they completed the entire screening procedure. Those who met the cognitive (SATURN) and hearing requirements were invited to participate in Session 2 – experimental phase.

##### Cognitive screening task

Participants underwent the Self-Administered Tasks Uncovering Risk of Neurodegeneration (SATURN), a free self-administered online test, used to exclude participants with potential cognitive impairment (Bissig et al., 2020; Tagliabue et al., 2023). The main domains assessed are: Attention, Orientation, and Memory.

SATURN has been validated against the Montreal Cognitive Assessment (MoCA), showing a strong correlation with MoCA scores (*r* = 0.90, *p* < 0.001; Bissig et al., 2020). Given that the commonly accepted cutoff for cognitive integrity on the MoCA is ≥ 26, we adopted a conservative criterion and included only participants scoring > 26 on the SATURN.

##### Headphone-use screening tests

To ensure participants’ compliance with wearing stereo headphones—critical for the auditory component of our study—we administered two online headphone-use screening tests. These tests aimed to exclude individuals using single-channel headphones/earphones or loudspeakers, as well as those with difficulty discriminating volume or pitch differences. Participants first adjusted their device volume to a comfortable level and were instructed not to make further adjustments. They then completed two screening tests:

1. Anti-Phase (AP) Test (Woods et al., 2017): A three-alternative forced-choice (3AFC) intensity-discrimination task in which participants identified the quietest of three 200-Hz tones (1000-ms duration). The tones included a standard, a target that was 6 dB softer than the standard, and a “foil” identical to the standard but with opposite polarity—creating a 180° phase difference between the left and right headphone channels (anti-phase tone). When played over loudspeakers, this phase difference reduces loudness, leading to misidentification of the target tone. The passing criterion was at least 5 correct responses out of 6, with prior validation showing a 100% pass rate for headphone users and only 18% for loudspeaker users.
2. Huggins Pitch (HP) Test (Milne et al., 2021): A 3AFC detection task in which participants identified which of the three white noise stimuli (1000-ms duration) contained a faint tonal percept. This illusory pitch arises only when white noise is presented to one ear and the same noise but in anti-phase (opposite polarity) is presented to the other ear. Because this effect relies on binaural processing, it is only perceivable with stereo headphones. With a cutoff of 5/6 correct trials, the test correctly identifies 80% of headphone users but has a 20% false-positive rate for loudspeaker users.

Each task included a familiarization phase, followed by six trials (∼2 minutes per task) without feedback. Participants were excluded in case they failed both tests.

#### Session 2 – experimental phase

On a separate day, participants completed a screen calibration procedure prior to the main task. Next, they performed an auditory and a visual oddball task (pre-intervention tasks; the task order was counterbalanced across participants). Afterwards, each participant was randomly assigned to one of three experimental groups (Placebo, Nocebo, Control; see Placebo/Nocebo intervention section), and received a written description of the expected effects of an upcoming acoustic intervention (see Supplementary Material for details). Participants were then exposed to a four-minute inert acoustic frequency. Immediately after the acoustic intervention and before completing the post-intervention oddball tasks they were asked to report their expectations regarding any potential change in cognitive performance due to the intervention (see Supplementary Materials for the exact wording of the question and the response format). Following this expectancy assessment, participants completed the oddball tasks again in both sensory modalities (post-intervention tasks; task order again counterbalanced across participants). At the end of the session, participants reported their perceived intervention efficacy, i.e., their subjective perception of whether their performance had changed after the sound intervention (see Supplementary Materials for the exact wording of the question and the response format).

All participants received a debriefing at the end of the session. They were informed of the true purpose of the study, the reason for the deceptive information, and the experimental group to which they had been assigned. The session lasted approximately 60 minutes.

##### Screen and volume calibration

To ensure that the visual stimuli were presented at a consistent pixel resolution relative to the participant’s screen dimensions, we estimated screen resolution using the Card Task (Li et al., 2020). Participants were instructed to sit at an arm’s length from the computer. They then placed a credit card (or a card of the same standardized size) on the screen and adjusted a slider until the on-screen image of the card matched the real-world card. This allowed us to calculate the logical pixel density (LPD) of the display in pixels per millimeter, enabling the precise presentation of stimuli in pixels (on-screen distance) regardless of individual screen sizes and resolutions. Participants were also asked to adjust their device volume to a comfortable level and were instructed not to make further adjustments for the rest of the experiment.

##### Adaptive oddball task

The task was delivered separately in the auditory and visual modalities, with the order of presentation counterbalanced across participants. There were two types of stimuli:

1. Standard Stimuli: Presented with a higher frequency (85% of the total trials).
2. Odd (Target) Stimuli: Presented with a lower frequency (15% of total trials).

Participants were instructed to press the space bar whenever the odd stimulus appeared and to ignore the frequent standard stimulus (Justen & Herbert, 2018; Sbaihat et al., 2021). To make the task challenging, while minimizing ceiling effects and accounting for individual differences in the aging population, the task was made adaptive: all participants started each session (PRE and POST) from the same initial level of difficulty, and the perceptual difference between the odd and standard stimuli was then dynamically adjusted based on the participant’s performance. Namely, the difficulty was regulated through an adaptive staircase procedure, where difficulty increased by one level after the correct execution of 14 consecutive trials, and decreased by one level following at least one mistake within a block of 7 consecutive trials. The trial order was pseudo-randomized to ensure that the sequence of standard and odd stimuli remained unpredictable, while maintaining the constraints that one odd (target) stimulus appeared within every set of 7 consecutive trials, and at least 2 standard trials separated each odd (target) stimulus to reduce anticipatory responses. The task consisted of 315 trials for each sensory modality, and the stimulus onset asynchrony (SOA) randomly varied between 850 ms and 1150 ms. Stimuli were displayed on a gray background (RGB values: 128, 128, 128). Before starting the main task, participants underwent 14 practice trials in which the odd stimulus appeared 3 times. During these practice trials, the perceptual difference between the odd and standard stimuli was kept constant at the largest difference. Written feedback was provided at the end of each practice trial.

##### Auditory version

**Fig. 1:**
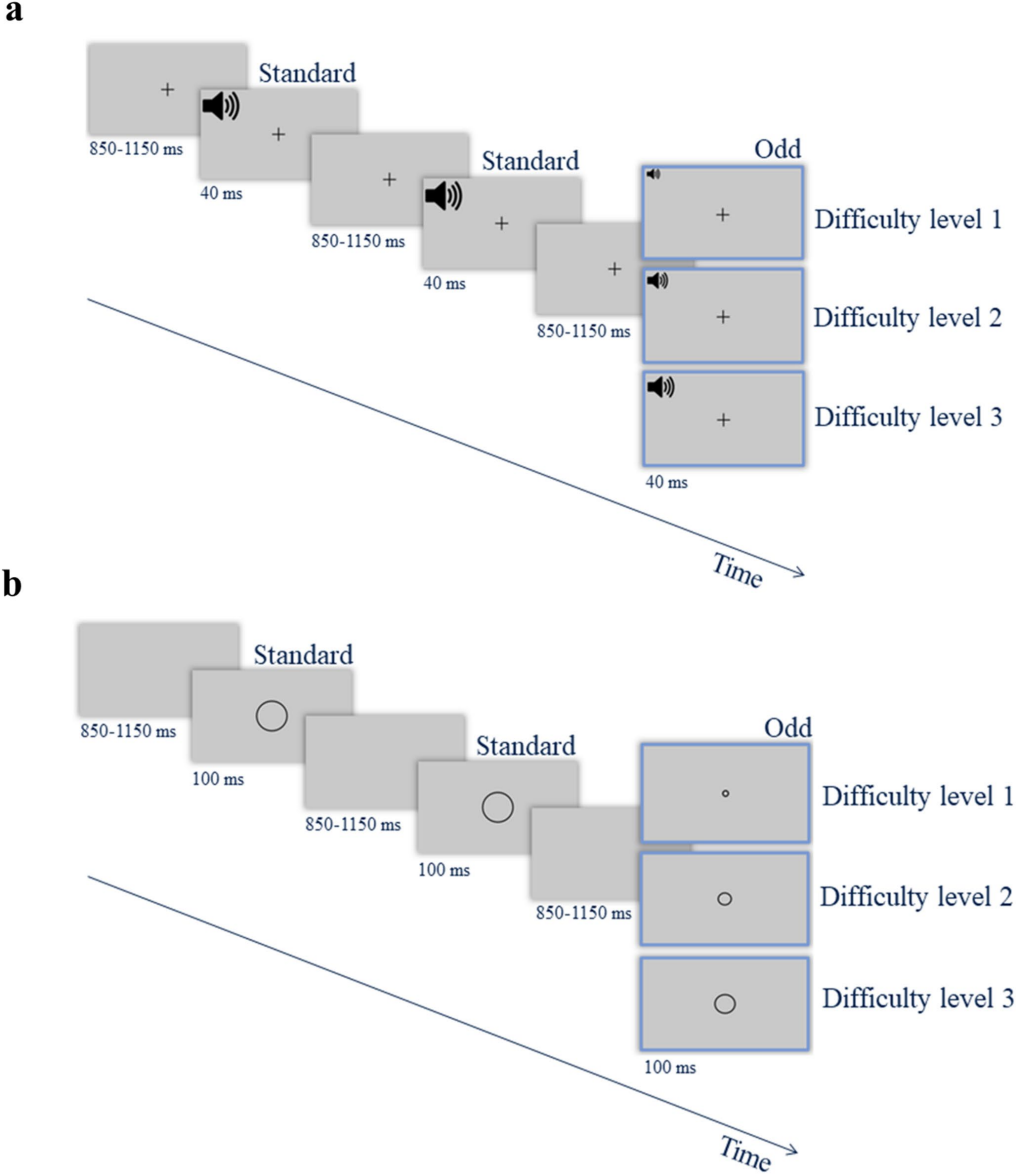
Experimental design. *Note:* *Panel a*: Auditory oddball task: Each trial began with a fixation cross that remained on the screen throughout the trial, followed by the presentation of a tone. The tone was either a standard stimulus with a fixed frequency or an odd (target) stimulus, whose frequency varied according to the participant’s performance. *Panel b*: Visual oddball task: Each trial began with a blank screen, followed by the presentation of a circle. The circle could be either a standard stimulus with always the same size or an odd (target) stimulus, whose size varied based on the achieved difficulty level.

In the Auditory Oddball Task (Fig. 1a), participants were required to discriminate between odd (target) and standard sound stimuli based on frequency. Both odd and standard stimuli were presented with a 40-ms duration and 4-ms fade-in and fade-out times to ensure smooth transitions. The standard stimulus had a fixed frequency of 870 Hz. The odd stimulus frequencies started at 970 Hz (the greatest perceptual difference) and could range down to 872 Hz. The auditory task had a total of 18 difficulty levels, each defined by the frequency difference between the odd and standard tones. The first nine levels decreased in 10 Hz steps, while the remaining levels decreased in finer 2 Hz steps. Therefore, as the difficulty increased, the frequency difference between the target and the standard stimuli decreased (i.e., making the target harder to detect).

##### Visual version

In the Visual Oddball Task (Fig. 1b), stimuli were circles of varying size, and participants were required to discriminate between odd (target) and standard circle stimuli based on size. Stimulus size was defined in degrees of visual angle, with actual dimensions adjusted to each participant’s screen resolution as determined during the screen calibration phase. The standard stimulus had a fixed size, which remained constant across all trials. In contrast, the odd (target) circles had a dynamically changing size controlled by a scaling factor, which adjusted according to the current difficulty level. The difficulty level ranged from 1 to 16, with difficulty level 1 corresponding to a scaling factor of 0.5 and difficulty level 16 corresponding to a scaling factor of 0.975. The criterion for defining difficulty levels was based on the size difference between the odd and standard stimuli: from level 1 to 5, the scaling factor increased in steps of 0.05; from level 6 to 16, the scaling factor increased in smaller, consistent steps of 0.025. As the difficulty increased, the difference between the size of the target and the standard stimulus became smaller (i.e., making the target more challenging to detect). Each stimulus was displayed for 100 milliseconds. The stimuli appeared at random positions on the screen within a specified range (± 0.25 degrees of visual angle) horizontally and vertically from the screen center to prevent location-based masking effects. After each stimulus presentation, the screen was briefly cleared.

##### Placebo/nocebo intervention

To investigate the influence of expectancy on performance, we implemented a placebo/nocebo intervention (Price et al., 2008; Colloca & Miller, 2011; Wager & Atlas, 2015; Enck et al., 2008). Specifically, participants in the Placebo group were exposed to a 4-minute pure tone at 240 Hz (actually inert) right after reading an informative script emphasizing the potential benefits of such intervention in inducing positive effects on attention and concentration. This specific acoustic sound has been previously used as a control condition in cognitive studies (Beauchene et al., 2016, 2017) with even longer durations. Furthermore, Wang et al. (2022) demonstrated that exposure to a 240 Hz tone does not affect brain connectivity or cognitive performance, confirming its suitability as an inert intervention. The Nocebo group was subjected to the same inert acoustic intervention and duration as the Placebo group. However, participants in the Nocebo group were given a written script that emphasized the adverse effects of the sound on cognitive performance, specifically attention and concentration. In contrast, the Control group received a neutral written script informing them about the inert nature of the acoustic sound. This allowed for an assessment based uniquely on their exposure to the auditory frequency.

### Statistical analyses

Statistical analyses were conducted using RStudio (version 4.3.2, R Core Team, 2023).

For the outlier removal, performance-based exclusions were carried out separately for each task (auditory and visual oddball) and for each session (PRE and POST intervention). A two-step procedure was applied:

1. Trial-level accuracy screening: Participants were excluded if they made errors on more than half of the standard trials (false alarms) or more than half of the odd (target) trials (i.e., misses or late responses). This step aimed to identify participants who, despite having completed standardized practice trials, did not appear to understand or properly engage with the task during the main session.
2. Distribution-based exclusion: Based on PRE-intervention performance only, we applied the interquartile range (IQR) method. Participants whose error rates exceeded third quartile (Q3 + 1.5 × IQR) for either standard or odd (target) trials were excluded. This step was limited to PRE to avoid excluding participants whose POST performance may have been influenced by the experimental manipulation.

This procedure ensured that final analyses were based on participants demonstrating reliable task engagement across both timepoints. The process was carried out separately for each sensory modality.

#### Demographic characteristics

To ensure comparability across experimental groups, one-way ANOVAs were conducted on key demographic variables separately for each condition (unimodal and crossmodal). The dependent variables included age, gender, years of education, SATURN score and performance on headphone-use screenings (i.e., the proportion of participants in each group who passed the AP test, the HP test, and both tests). In each analysis, Group was entered as a between-subjects factor with three levels: Control, Placebo, Nocebo.

#### The placebo and nocebo effects on performance in oddball tasks

The first analysis evaluated the placebo and nocebo effects on performance in the oddball tasks. Rather than relying on a single summary metric (e.g., the overall mean or maximum difficulty level for the PRE and POST performances), we modeled performance trends across trials to capture the trajectory of learning or adaptation within each performance. Considering trial-by-trial changes in difficulty level as performance measure allowed us to assess not only overall performance but also how it evolved over time. Modeling Trial as a continuous variable enabled the detection of subtle changes in performance trend that might be obscured by aggregate indices. Therefore, the overall design always included Group as a between-subjects factor (three levels: Control, Nocebo, Placebo) and Trial (continuous) as a within-subject factor. Nonetheless, we reported overall mean or maximum difficulty level for PRE and POST performances in Supplementary Materials.

Preliminary analyses assessed group comparability in PRE (baseline) performance by analysing trial-by-trial trends in difficulty level. A linear mixed-effects model was used, with difficulty level as the dependent variable, Trial and Group as fixed-effects, and Participant as a random effect. In this model, the estimated slope associated with trial represents the linear trend of performance across trials within each group, indicating whether performance tended to improve or decline over time. Given the significant differences found between groups at PRE (see Supplementary Materials), we opted to use the delta (i.e., POST minus PRE) of trial-by-trial difficulty level as the primary performance measure. By analyzing the difference in linear trial-wise trends between POST and PRE, we were able to more effectively isolate the effects of the experimental manipulation, account for baseline variability between groups, and reduce measurement noise—thereby increasing statistical power.

A linear mixed-effects model was fitted to analyze differences in trial-by-trial difficulty level trends between POST- and PRE-intervention performance. Participant was considered as a random effect. We conducted a Type III ANOVA on the model effects and computed effect sizes (partial eta-squared). Significant Group main effects as well as Group-by-Trial interactions were further examined through post-hoc tests with Bonferroni correction applied for multiple comparisons. Additionally, to determine whether the delta of trial-by-trial difficulty level linear trend was significantly different from zero within each group, we conducted further post-hoc analyses, again applying Bonferroni correction for both confidence intervals and p-values.

#### The placebo and nocebo effects on subjective expectations and perceived intervention efficacy

The second analysis investigated the effect of positive and negative verbal suggestions on the participants’ subjective expectancy and perceived efficacy of the intervention. Here, the reported expectancy was treated as a dependent variable with three levels (“Expectancy of worsening”, “Expectancy of no change”, “Expectancy of improvement”), and the reported perceived efficacy of the intervention was treated as a dependent variable with three levels (“Perception of worsening”, “Perception of no change”, “Perception of improvement”). The overall design included Group as between-subjects factor with three levels (Control, Nocebo, Placebo). Because the data consisted of frequency counts for each category, and some cells had very small expected values, Fisher’s Exact Test was performed to examine whether the distribution of expectancy and perceived intervention efficacy ratings differed across the three experimental groups. When significant effects were detected, post-hoc pairwise comparisons were carried out with Bonferroni correction applied to adjust for multiple testing. These post-hoc tests were structured as 2 × 2 Fisher’s Exact Tests, comparing each pair of groups across each pairwise combination of expectancy levels.

## Results

### Participants

Of the total 204 older adults initially recruited for Session 1, only 177 participants completed Session 1 after accounting for pilot testing, technical issues, and returned submissions. From this sample, 76 participants were excluded based on pre-screening criteria, cognitive performance (SATURN), or headphone-use screenings, leaving 101 older adults invited to participate in Session 2 – experimental phase. Of these, 96 participants completed Session 2.

**Fig. 2:**
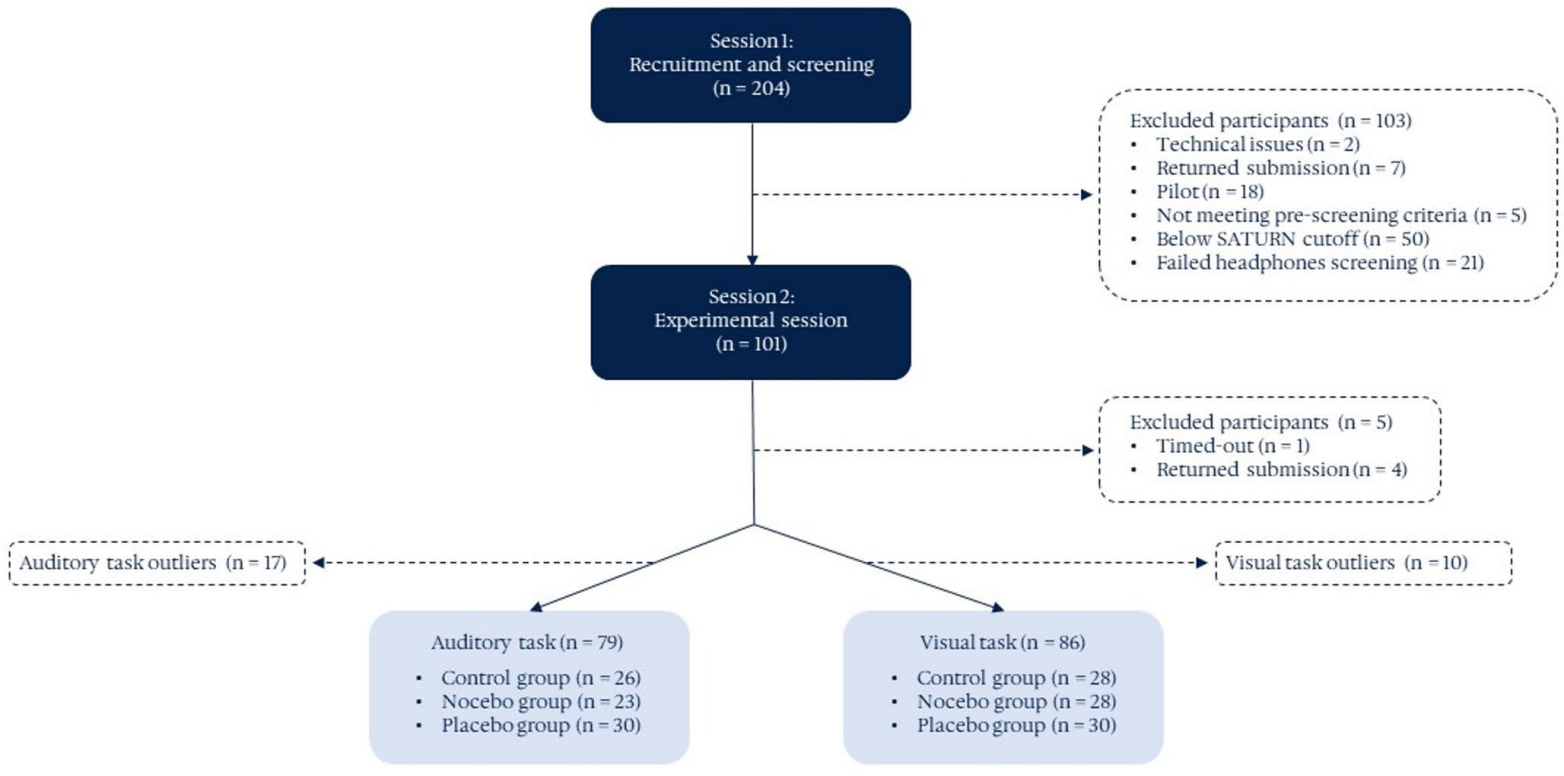
Flowchart illustrating the flow of participants through each phase of the study. Note: The flowchart displays step-by-step sample sizes from initial recruitment through eligibililty checks, and outlier removal, leading to the final group sizes for both the auditory and visual oddball tasks.

Following the completion of Session 2, performance-based exclusions were applied. A two-step outlier removal procedure was implemented separately for the auditory and visual oddball tasks (see Statistical Analyses section), resulting in final samples of 79 (mean age = 68.44, *SD* = 3.65, 37 females) and 86 older adults (mean age = 68.84, *SD* = 3.56, 45 females), respectively. A detailed overview of participant recruitment, exclusions, and group allocation is provided in Fig. 2, and descriptive statistics for participants’ sociodemographic characteristics and screening measures are presented in Table 1.

**Table 1:** Descriptive statistics of participants’ characteristics and screening measures included for the oddball tasks.

	Auditory			Visual		
	Control group	Nocebo group	Placebo group	Control group	Nocebo group	Placebo group
	*n* = 26	*n* = 23	*n* = 30	*n* = 28	*n* = 28	*n* = 30
Age (years)	69.08 ± 4.12 Range (65–78)	68.52 ± 3.19 Range (65–75)	67.97 ± 3.42 Range (65–79)	69.21 ± 3.97 Range (65–78)	68.75 ± 3.07 Range (65–75)	68.6 ± 3.63 Range (65–79)
Biological sex (F/M)	6/20	13/10	18/12	7/21	17/11	21/9
Education (years)	15.35 ± 3.59 Range (10–23)	15.91 ± 3.1 Range (12–24)	15 ± 2.51 Range (11–20)	15.64 ± 3.58 Range (10–23)	15.25 ± 3.27 Range (9–24)	14.8 ± 2.65 Range (11–20)
SATURN	27.96 ± 0.77	28.52 ± 0.73	28.23 ± 0.77	28.04 ± 0.75	28.46 ± 0.69	28.23 ± 0.82
HS: combined pass rate	73.08%	73.91%	66.67%	75%	71.43%	66.67%

HS = headphone-use screening tests. Values for Age, Education, and SATURN are reported as mean ± standard deviation. Biological sex is reported as number of females/males. For headphone-use screening tests, the combined pass rate reported refers to the percentage of participants who passed both screening tests

### Demographic characteristics and screening measures

In the unimodal condition, the groups resulted non-homogeneous in terms of gender and SATURN scores, whereas in the crossmodal condition, the groups significantly differed only in gender (see Supplementary Materials).

### The placebo and nocebo effects on performance in oddball tasks

#### Unimodal condition

**Fig. 3:**
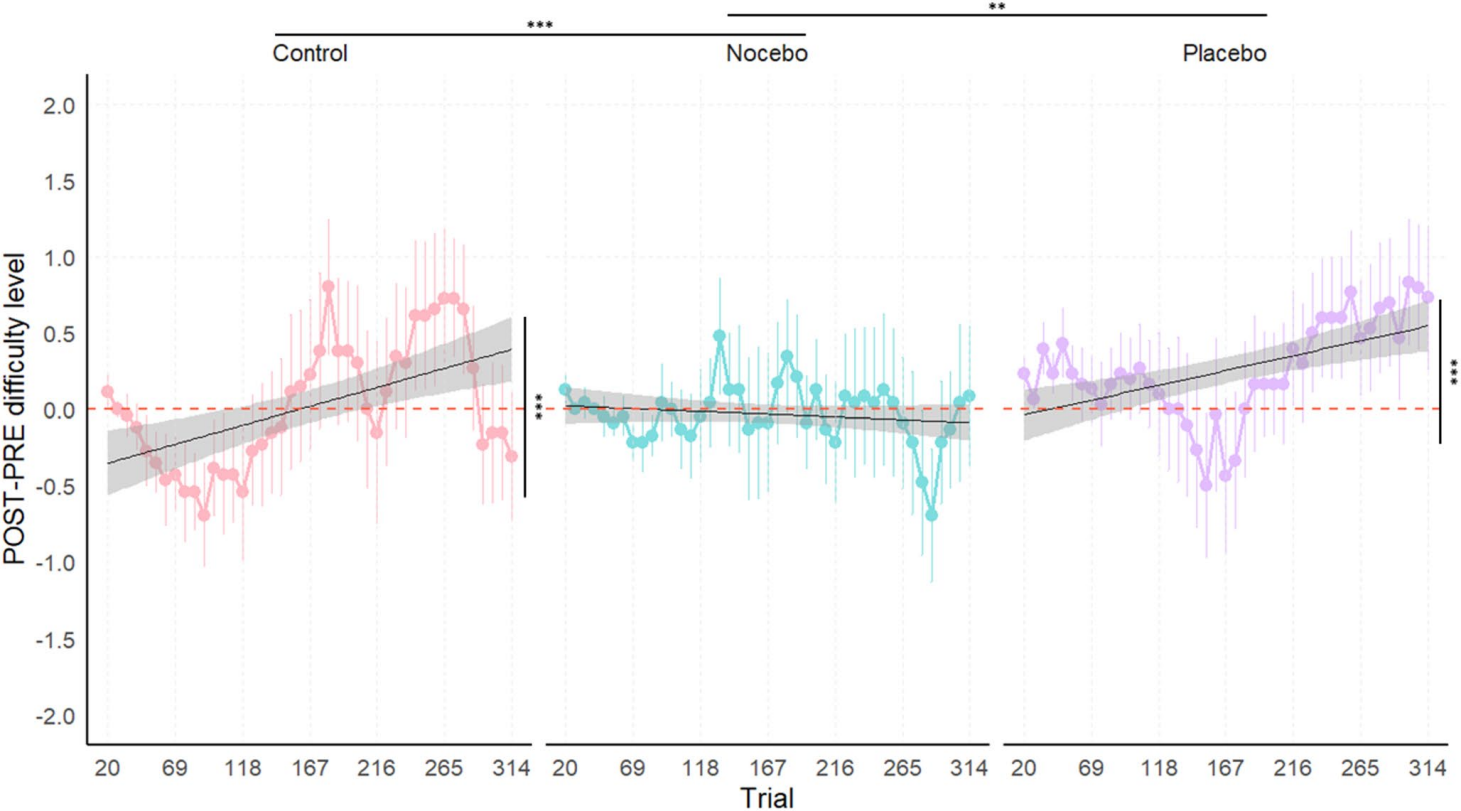
Auditory oddball task – Delta (POST – PRE) of trial-by-trial difficulty level across groups. Note: Positive values of the delta indicate improved performance, while negative values reflect a decline in performance following the intervention (POST) compared to baseline (PRE). Each panel includes a linear regression line (black) depicting the trial-by-trial trend of performance within each group; shaded bands denote the 95% confidence interval around the fitted line. Significance codes: ‘***’ for p < 0.001, ‘**’ for p < 0.01.

In the auditory oddball task (see Fig. 3), the marginal ANOVA of the linear mixed model of the delta of trial-by-trial difficulty level trends revealed a significant main effect of Trial, *F*(1, 3315) = 22.59, *p* < 0.001, η^2^_p_ = 0.006, indicating that the change in performance trends across trials between PRE and POST was significant. The main effect of Group was not significant, *F*(2, 97.46) = 0.73, *p* = 0.485, η^2^_p_ = 0.01. However, the significant Trial x Group interaction, *F*(2, 3315) = 7.831, *p* < 0.001, η^2^_p_ = 0.005, indicated that the change in trial-by-trial difficulty level trends from PRE to POST differed across groups.

To further investigate the interaction, post-hoc pairwise comparisons of the delta of trial-by-trial difficulty level trends between groups revealed significant differences between the Control and Nocebo groups, *t*(3315) = 3.735, *p* < 0.001, as well as between the Nocebo and Placebo groups, *t*(3315) = −3.141, *p* = 0.005. The difference between the Control and Placebo groups was not significant, *t*(3315) = 0.741, *p* = 1. These results indicate that the Nocebo group exhibited a distinct pattern of change in performance trends across trials from PRE to POST compared to the other groups.

Trend analyses within each group showed a significant positive delta of trial-by-trial difficulty level trends indicating improvement in performance for the Control, *t*(3315) = 4.753, *p* < 0.001, and Placebo, *t*(3315) = 4.018, *p* < 0.001, groups, with increasing difficulty levels across trials at POST relative to baseline. In contrast, the delta of trial-by-trial difficulty level trends of the Nocebo group did not significantly differ from zero, *t*(3315) = −0.657, *p* = 1. The improvement observed in the Control group likely reflects a practice effect, suggesting that the lack of such an effect in the Nocebo group may indicate the effectiveness of the negative verbal suggestion in counteracting performance gains typically associated with repeated task exposure.

#### Crossmodal condition

**Fig. 4:**
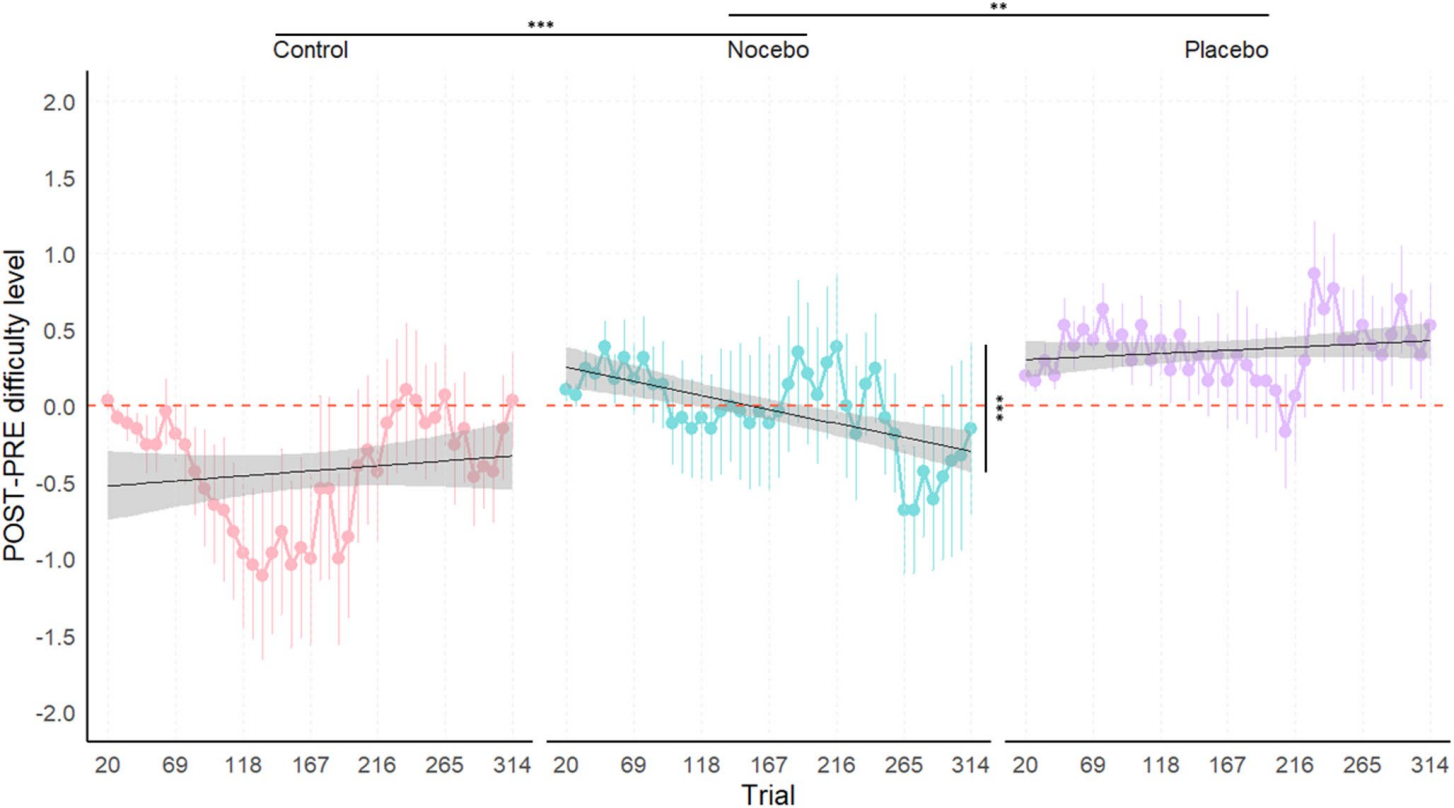
Visual oddball task – Delta (POST – PRE) of trial-by-trial difficulty level across groups. Note: Positive values of the delta indicate improved performance, while negative values reflect a decline in performance following the intervention (POST) compared to baseline (PRE). Each panel includes a linear regression line (black) depicting the trial-by-trial trend of performance within each group; shaded bands denote the 95% confidence interval around the fitted line. Significance codes: ‘***’ for *p* < 0.001, ‘**’ for *p* < 0.01.

In the visual oddball task (see Fig. 4), the marginal ANOVA of the linear mixed model analysis of the delta of trial-by-trial difficulty level trends revealed no significant main effect of Trial, *F*(1,3609) = 1.993, *p* = 0.158, η^2^_p_ < 0.001. The main effect of Group was significant, *F*(2,100.244) = 3.296, *p* = 0.041, η^2^_p_ = 0.062, although no significant differences emerged from the post-hoc analyses (*p* > 0.05). In addition, a significant Trial x Group interaction, *F*(2,3609) = 8.743, *p* < 0.001, η^2^_p_ = 0.005, indicated that the delta of trial-by-trial difficulty level trends differed across groups.

To further investigate the significant interaction, post-hoc pairwise comparisons of the delta of trial-by-trial difficulty level trends between groups revealed significant differences between the Control and Nocebo groups, *t*(3609) = 3.778, *p* < 0.001, as well as between the Nocebo and Placebo groups, *t*(3609) = −3.465, *p* = 0.002. The difference between the Control and Placebo groups was not significant, *t*(3609) = 0.377, *p* = 1.

Trend analyses within each group showed a significant negative delta of trial-by-trial difficulty level trends for the Nocebo group, *t*(3609) = −4.93, *p* < 0.001, indicating a decrease in the difficulty level across trials at POST-intervention relative to baseline. In contrast, the delta of trial-by-trial difficulty level trends of the Control and Placebo groups did not significantly differ from zero, *t*(3609) = 1.412, *p* = 0.474, and *t*(3609) = 0.919, *p* = 1, respectively.

### Post-hoc analyses on placebo and nocebo believers

To specifically assess the impact of positive and negative expectancies on performance in the oddball tasks, we conducted post-hoc analyses by stratifying participants from the Placebo and Nocebo groups based on the congruency between the verbal suggestion received and reported (subjective) expectation. This resulted in 14 Placebo believers (i.e., participants who received positive verbal suggestions and reported an improvement expectancy) and 6 Nocebo believers (i.e., participants who received negative verbal suggestions and reported a worsening expectancy) in the unimodal condition, and 12 Placebo believers and 7 Nocebo believers in the crossmodal condition.

#### The placebo and nocebo effects on Placebo and Nocebo believers’ performance in oddball tasks

The statistical approach mirrored that of the main analysis, using the delta of trial-by-trial difficulty level trends as the primary performance index due to the significant PRE-intervention differences in performance trajectories between the two believer groups in both modalities (see Supplementary Materials). In this focused comparison, the Group factor included only two levels: Placebo believers and Nocebo believers.

#### Unimodal condition

**Fig. 5:**
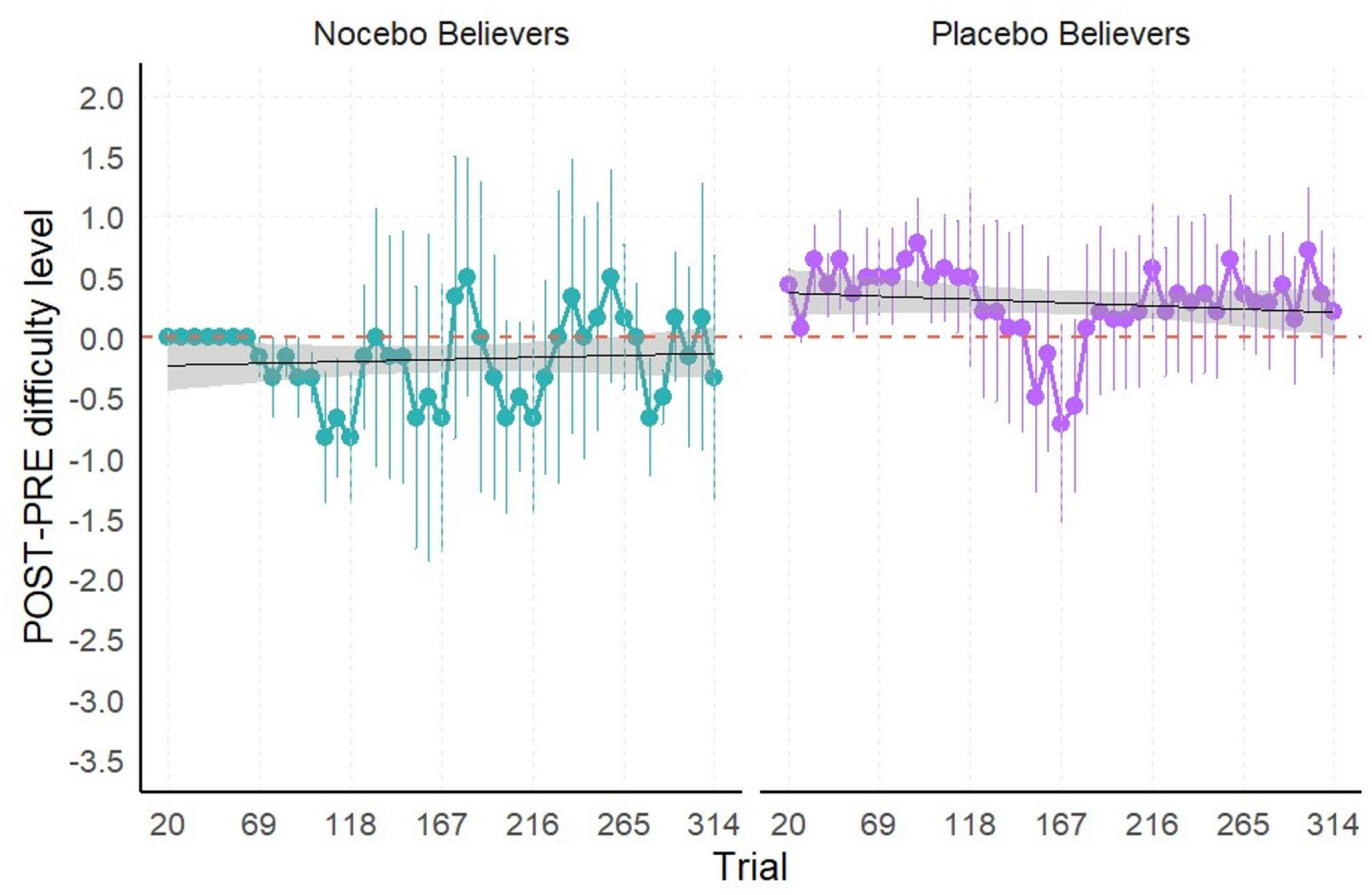
Auditory oddball task – Delta (POST – PRE) of trial-by-trial difficulty level across the believer groups. Note: Positive values of the delta indicate improved performance, while negative values reflect a decline in performance following the intervention (POST) compared to baseline (PRE).

The marginal ANOVA of the linear mixed-effects model analyzing the delta of trial-by-trial difficulty level trends (see Fig. 5) indicated no significant main effect of Trial, *F*(1,838) = 0.083, *p* = 0.773, η^2^_p_ < 0.001, Group, *F*(1,24.583) = 0.993, *p* = 0.329, η^2^_p_ = 0.039, or the interaction between Trial and Group, *F*(1,838) = 0.394, *p* = 0.53, η^2^_p_ < 0.001.

Trend analyses within each group showed that the delta of trial-by-trial difficulty level trends did not significantly differ from zero for either group: *t*(838) = 0.288, *p* = 0.773 for the Nocebo Believers, and *t*(838) = −0.705, *p* = 0.481 for the Placebo Believers.

#### Crossmodal condition

**Fig. 6:**
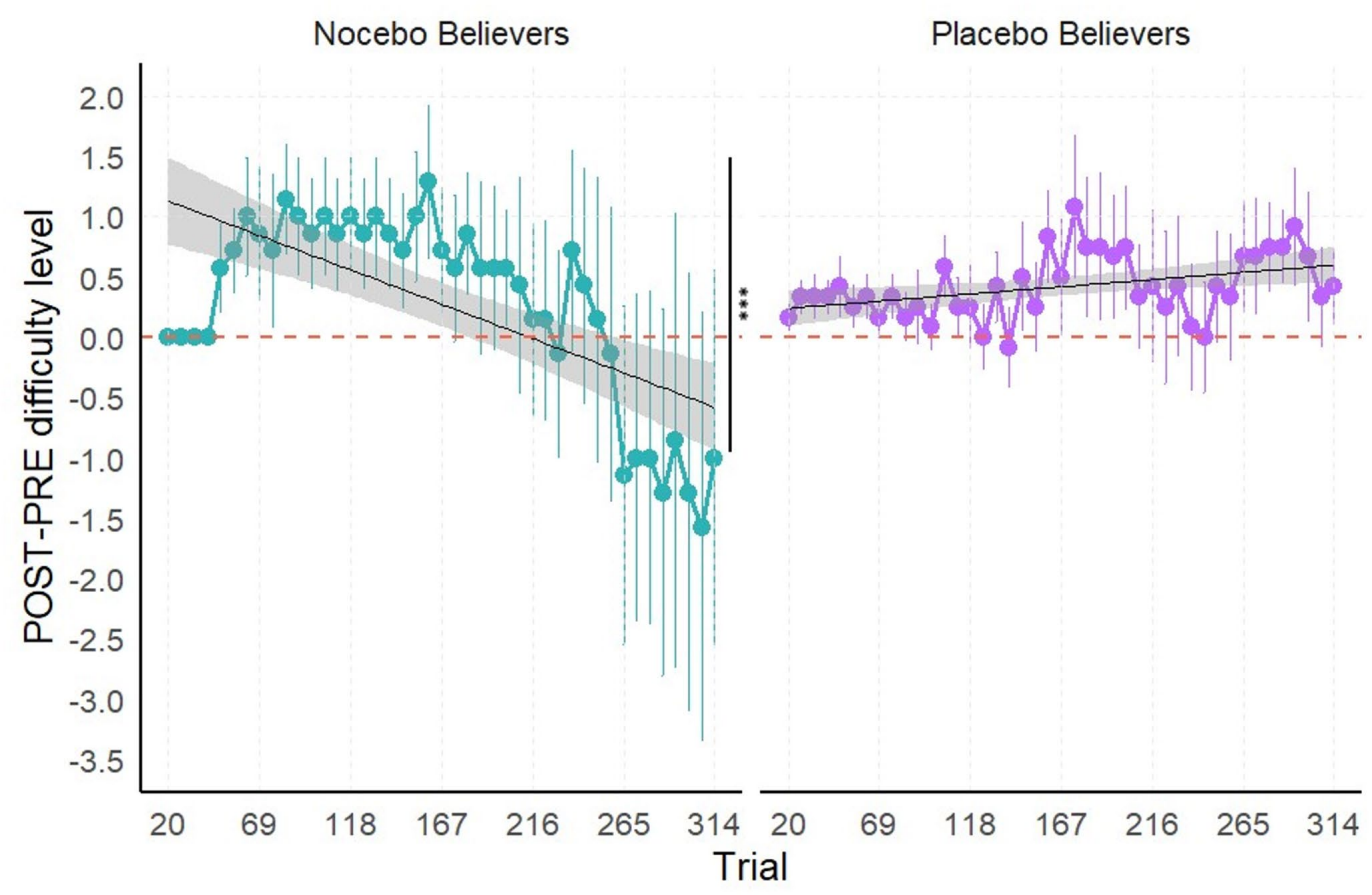
Visual oddball task – Delta (POST – PRE) of trial-by-trial difficulty level across the believer groups. Note: Positive values of the delta indicate improved performance, while negative values reflect a decline in performance following the intervention (POST) compared to baseline (PRE). Significance code: ‘***’ for *p* < 0.001.

The marginal ANOVA of the linear mixed-effects model of the delta of trial-by-trial difficulty level trends (see Fig. 6) revealed a significant main effect of Trial, *F*(1,796) = 35.02, *p* < 0.001, η^2^_p_ = 0.017, indicating a significant change in performance trends across trials from PRE- to POST-intervention. The main effect of Group showed a tendency towards significance, *F*(1,23.689) = 3.806, *p* = 0.063, η^2^_p_ = 0.138. Additionally, a significant interaction between Trial and Group was found, *F*(1,796) = 32.333, *p* < 0.001, η^2^_p_ = 0.039.

Trend analyses showed a significant negative delta of trial-by-trial difficulty level trends for the Nocebo believers, *t*(796) = −5.918, *p* < 0.001, indicating a decrease in the difficulty level across trials at POST-intervention relative to baseline. In contrast, the delta of trial-by-trial difficulty level trends of the Placebo believers did not significantly differ from zero, *t*(796) = 1.62, *p* = 0.106, suggesting that they did not exhibit a significant practice effect in the POST-intervention performance.

### The placebo and nocebo effects on subjective expectations

#### Unimodal condition

The results of the Fisher’s Exact Test indicated a significant association between group assignment and reported expectations, *p* <.001 (see Table 2 for the absolute and relative frequencies of expectancy endorsements across groups). Post-hoc pairwise comparisons revealed a significant difference between the Control and Placebo groups when comparing both “Expectancy of improvement” vs. “Expectancy of no change”, *p* < 0.001, and “Expectancy of improvement” vs. “Expectancy of worsening”, *p* = 0.015; no significant difference emerged between “Expectancy of worsening” and “Expectancy of no change”, *p* = 1. Although approximately half of the participants in the Placebo group still reported expecting no change, a notably larger proportion (46.7%) reported expecting improvement compared to the Control group (0%), suggesting that Placebo participants were substantially more inclined to anticipate an improvement in their performance than those in the Control group. This pattern indicates that the verbal suggestion was effective in increasing improvement-related expectancies among Placebo participants.

Since neither group included participants who reported expecting improvement, the comparison between the Control and Nocebo groups focused on expectations of worsening versus no change, and no significant difference was observed in their distribution, *p* = 0.27. Additional analyses, including aggregated and numerical scoring approaches, yielded convergent results (see Supplementary Materials).

#### Crossmodal condition

The results of the Fisher’s Exact Test indicated a significant association between group assignment and reported expectancy, *p* < 0.001 (see Table 2 for the absolute and relative frequencies of expectancy endorsements across groups). Post-hoc pairwise comparisons revealed a significant difference between the Control and Placebo groups only when comparing both “Expectancy of improvement” vs. “Expectancy of no change”, *p* = 0.015, and no significant difference emerged between “Expectancy of improvement” vs. “Expectancy of worsening”, *p* = 0.253, or between “Expectancy of worsening” and “Expectancy of no change”, *p* = 1. Although approximately half of the participants in the Placebo group reported expecting no change, a notably larger proportion (40%) reported expecting improvement compared to the Control group (7.1%), suggesting that they were overall more likely than those in the Control group to expect improvement. This pattern indicates that the verbal suggestion was effective in increasing improvement-related expectancies among Placebo participants.

The comparison between the Control and Nocebo groups revealed no significant differences in any expectancy level, with all *p*s > 0.05. Additional analyses, including aggregated and numerical scoring approaches, yielded convergent results (see Supplementary Materials).

**Table 2:** Auditory and visual oddball tasks – Absolute frequencies of expectancy endorsements by participants in each group.

		Expectancy of worsening	No change expected	Expectancy of improvement	Total
Auditory					
Control		3 (11.5%)	23 (88.5%)	0	26
Nocebo		6 (26.1%)	17 (73.9%)	0	23
Placebo		1 (3.3%)	15 (50%)	14 (46.7%)	30
** Total**		10	55	14	79
Visual					
Control		3 (10.7%)	23 (82.1%)	2 (7.1%)	28
Nocebo		7 (25%)	21 (75%)	0	28
Placebo		2 (6.7%)	16 (53.3%)	12 (40%)	30
** Total**		12	60	14	86

Relative frequencies are shown in parentheses and represent within-group proportions (i.e., calculated as percentages of the total number of participants in each group).

### The placebo and nocebo effects on subjective level of perceived intervention efficacy

#### Unimodal condition

The results of the Fisher’s Exact Test indicated a significant association between group assignment and perceived efficacy of the intervention, *p* = 0.015 (see Table 3 for the absolute and relative frequencies of perceived efficacy endorsements across groups). Post-hoc pairwise comparisons revealed a significant difference between the Control and Placebo groups specifically in the distribution of “Perception of improvement” versus “Perception of worsening”, *p* = 0.019. While approximately half of the Control group and the majority of the Placebo group reported to have not perceived a change in their performance, fewer participants in the Placebo group reported a perceived worsening (10%) compared to the Control group (42.3%). Conversely, a greater proportion of Placebo participants (23.3%) reported perceiving an improvement in their performance compared to only 3.8% in the Control group. These patterns suggest that Placebo participants were more inclined to perceive an improvement and less likely to report a decline in performance.

The comparison between the Control and Nocebo groups and the Placebo and Nocebo groups revealed no significant differences in any level of perceived efficacy, all *p*s > 0.05. Additional analyses, including aggregated and numerical scoring approaches, yielded convergent results (see Supplementary Materials).

#### Crossmodal condition

The results of the Fisher’s Exact Test indicated a significant association between group assignment and perceived efficacy of the intervention, *p* = 0.014 (see Table 3 for the absolute and relative frequencies of perceived efficacy endorsements across groups). Post-hoc pairwise comparisons revealed a significant difference between the Control and Placebo groups when comparing “Perception of improvement” versus “Perception of worsening”, *p* = 0.045, and “Perception of worsening” and “Perception of no change”, *p* = 0.044; no significant difference emerged between “Perception of improvement” vs. “Perception of no change”, *p* = 1. Although approximately half of the Control group and the majority of the Placebo group reported to have not perceived a change in their performance, the absolute and relative frequencies suggest distinct patterns between the groups. Specifically, fewer participants in the Placebo group reported a perceived worsening (10%) compared to the Control group (42.9%). Conversely, a larger proportion of Placebo participants (26.7%) reported perceiving an improvement than those in the Control group (10.7%). These patterns suggest that Placebo participants were more inclined to perceive an improvement and less likely to report a decline in performance.

The comparison between the Control and Nocebo groups as well as the Placebo and Nocebo groups revealed no significant differences in any level of perceived efficacy, with all *p*s > 0.05. Additional analyses, including aggregated and numerical scoring approaches, yielded convergent results (see Supplementary Materials).

**Table 3:** Auditory and visual oddball tasks – Absolute frequencies of perceived efficacy endorsements by participants in each group.

		Perception of worsening	No change perceived		Perception of improvement		Total
Auditory							
Control		11 (42.3%)	14 (53.8%)		1 (3.8%)		26
Nocebo		5 (21.7%)	17 (73.9%)		1 (4.3%)		23
Placebo		3 (10%)	20 (66.7%)		7 (23.3%)		30
** Total**		19	51		9		79
Visual							
Control		12 (42.9%)	13 (46.4%)		3 (10.7%)		28
Nocebo		5 (17.9%)	21 (75%)		2 (7.1%)		28
Placebo		3 (10%)	19 (63.3%)		8 (26.7%)		30
** Total**		20	53		13		86

Relative frequencies are shown in parentheses and represent within-group proportions (i.e., calculated as percentages of the total number of participants in each group)

## Discussion

The study investigated the impact of expectancy on changes in performance in a perceptual and attentional task undertaken by older individuals in an online setting. To explore this, we explicitly manipulated verbal suggestion regarding the effect of an (inert) acoustic intervention on performance in an oddball task, and additionally collected measures of subjective expectancy and perceived efficacy. Overall, the results revealed a dissociation: while the nocebo manipulation produced objective performance changes without corresponding shifts in subjective expectancy and perceived intervention efficacy, the placebo manipulation led to changes in subjective beliefs without measurable performance effects.

When examining the objective changes in performance, the findings for the Nocebo group indicate that the verbal suggestions influenced cognitive performance across both sensory modalities. Indeed, the results related to the auditory oddball task—the same modality as the intervention—indicated that the Nocebo participants remained stable, failing to exhibit the practice effect that was observed in the Control and Placebo groups. This suggests that the nocebo manipulation may have impaired learning processes, not through a direct decline in performance, but by preventing the typical practice-related improvement associated with task repetition. Conversely, in the visual task—where the modality differed from the intervention—the Nocebo group performed worse at POST, further supporting the presence of a nocebo effect, while the Placebo and Control groups showed no significant changes. Importantly, across both tasks, performance in the Placebo group did not significantly differ from the Control group, indicating no clear objective placebo effect.

Altogether, the Nocebo group consistently underperformed relative to the Control and Placebo groups. Therefore, these results point out that negative verbal suggestion consistently disadvantaged performance and were effective even when the sensory modality of the task differed from that of the intervention. This suggests that, despite differing in expression, the nocebo effect is relatively independent of sensory modality congruence.

Given the absence of an objective placebo effect at the group level, we conducted post-hoc analyses focusing on the subsample of participants whose expectations aligned with the intended manipulation—specifically, those in the Placebo group who expected improvement and those in the Nocebo group who expected worsening (i.e., Placebo and Nocebo believers). This approach allowed us to isolate the “ideal” conditions under which expectancy effects would most likely emerge. Although based on small and unbalanced sample sizes—and thus to be interpreted with caution—the post-hoc analyses revealed a nocebo effect for the visual oddball task, and no placebo benefit in either sensory modality. Notably, fewer than half of the Nocebo participants explicitly reported negative expectations. This may suggest that negative verbal suggestions influenced performance without necessarily translating into consciously endorsed expectations, pointing to a potential dissociation between objective and subjective processes.

Finding objective nocebo but not placebo effects raises questions about the mechanisms underlying these effects. Two aspects are particularly relevant and worth considering. First, research has predominantly focused on cognitive placebo effects, often neglecting the investigation of the nocebo counterparts. Even among the studies examining placebo effects on cognitive performance, findings have been inconsistent, with some reporting improvements (e.g., Colagiuri & Boakes, 2010; Oken et al., 2008; Turi et al., 2017), while others (e.g., Looby & Earleywine, 2011) have not. Fewer studies have investigated both placebo and nocebo effects within the same paradigm, and results have also been mixed. While some studies have reported clear evidence for both effects (e.g., Colagiuri et al., 2011; Turi et al., 2018), others have failed to find a measurable impact on cognitive performance (e.g., Winkler & Hermann, 2019; Schwarz & Buchel, 2015; Blokland, 2023). Overall, this pattern highlights a limited and inconclusive evidence base regarding nocebo effects in cognition.

Second, participant characteristics may have played a key role in shaping the present findings. The influence of aging on placebo responsiveness has received some attention in the broader placebo literature, particularly in the domain of pain. Although research specifically targeting older adults remains limited, available findings are mixed, with some studies suggesting that placebo effects can be preserved in older individuals (e.g., Bingel et al., 2011; Wrobel et al., 2016; Weimer et al., 2015), whereas others report reduced responses in older samples (e.g., Ho et al., 2009; Rischer et al., 2025). However, in the cognitive domain, most experimental studies have primarily involved young adults, whereas the sample in the present study consisted of older adults, who may be particularly susceptible to nocebo effects due to individuals’ self-perception of their aging and strong concerns about cognitive decline (Carbone et al., 2024). Evidence from the age-related stereotype-threat literature shows that activating negative aging stereotypes can impair cognitive performance in older adults (e.g., Barber & Mather, 2014; Hess et al., 2004, 2009), and although these mechanisms differ from expectancy-based placebo and nocebo effects, they nonetheless highlight how internalized views of aging may create a context in which negative suggestions resonate more strongly than positive ones, especially in older adults. In this sense, negative predictions may align with older adults’ existing beliefs about cognitive decline, whereas positive suggestions may have less psychological leverage in the absence of strong validating cues. In our case, it is plausible that the negative prediction about the intervention effects suggested in our study resonated with these pre-existing concerns about cognitive aging, thereby strengthening nocebo effects. At the same time, positive suggestions may have lacked sufficient psychological leverage to foster observable cognitive improvements (i.e., a placebo effect). This dynamic could have been magnified by the absence of direct social cues (due to the online procedure) within a population that may already struggle to achieve cognitive gains because of self-perceived biases on aging. Future studies incorporating measures of self-perceived aging, internalized aging stereotypes, or baseline self-assessments of cognitive ability could clarify how these processes interact in shaping cognitive performance in later life and whether individuals’ prior beliefs about their cognitive functioning predict responsiveness to placebo and nocebo effects.

When examining the subjective effects of our manipulation, namely the effect of the verbal suggestion on participants’ expectations regarding the impact of the intervention on their performance, we found that—despite most participants in both the Nocebo and Control groups, and about half of those in the Placebo group, were anticipating no change—verbal suggestions still influenced subjective reports in the Placebo group. Indeed, these participants were more likely to expect cognitive improvements across both sensory modalities. We also assessed whether the verbal suggestion affected participants’ perceived efficacy of the intervention. In this regard, a significant pattern emerged: participants in the Placebo group were more likely than those in the Control group to perceive an improvement in their cognitive performance following the inert acoustic intervention. This suggests that positive expectations not only influenced anticipated outcomes but also shaped retrospective perceptions of efficacy. Conversely, no significant effects were observed in the Nocebo group.

Taken together, the present findings reveal a dissociation between subjective beliefs and objective performance. On the one hand, verbal suggestion successfully influenced expectancy and perceived efficacy reports in the Placebo participants. Still, this effect did not translate into measurable performance improvements (beyond a practice effect that was also achieved by the Control group). This pattern mirrors findings from previous studies (Blokland, 2023; Schwarz & Büchel, 2015; Winkler & Hermann, 2019), which also reported that placebo-like manipulations effectively altered participants’ expectations and self-reported beliefs without producing corresponding changes in objective cognitive performance. We speculate that, particularly in cognitive domains, expectancy-induced subjective changes may not always be sufficient to elicit behavioral improvements, highlighting a potential boundary condition for placebo effects on cognition.

On the other hand, the Nocebo group did not exhibit a negative shift in either subjective expectancy or perceived efficacy, despite an objective nocebo response. This dissociative pattern echoes findings from Turi et al. (2017). In this study conducted in presence on young adults, results indicated an objective (placebo) effect following a sham NIBS intervention accompanied by placebo-inducing instructions without corresponding changes in participants’ subjective reports of expected or perceived cognitive enhancement. Performance benefits were modulated by the degree of uncertainty conveyed in the instructions, yet participants did not consciously attribute improvements to the intervention, suggesting that expectancy can operate implicitly or outside conscious awareness. Similarly, in our study, negative verbal suggestions may have influenced performance through more implicit mechanisms, such as self-efficacy beliefs or stable trait factors, rather than through consciously reported expectations. These implicit effects could have led participants to disengage from the task or perceive it as more aversive or tiresome, resulting in reduced effort, thereby contributing to the observed performance decrements. Although the contextual (online vs. in-person) and demographic (older vs. younger adults) differences limit direct comparisons, both studies highlight a dissociation between objective cognitive changes and consciously endorsed expectations. Future research could incorporate implicit measures of expectancy (e.g., MT-PEP; Cummins et al., 2024) to better capture these subconscious influences on performance.

Overall, the present findings suggest that, although expectancy remains a well-recognized mechanism underlying placebo and nocebo effects, it likely operates within a more complex interplay of mechanisms that may work together to shape subjective experience and objective performance. Consciously endorsed expectation likely operates in tandem with a broader set of cognitive, affective, and situational influences—such as motivation, engagement with the task, pre-existing beliefs, prior experience and personality traits—that collectively shape both perceived and actual cognitive outcomes (Colloca & Miller, 2011; Price et al., 2008; Wager & Atlas, 2015). The online nature of our study, which lacked direct interpersonal dynamics and experimenter presence, may have further attenuated contextual cues—such as experimenter bias—that typically support placebo effects. Improving cognitive performance may therefore require more than expecting to do well—it likely demands a degree of effortful engagement and cognitive investment that a brief verbal suggestion alone may not produce. In this respect, both the nature of the task and the strength of the placebo manipulation likely contributed to the limited observable effects. Tasks that rely more on automatic processing, such as the one used here, involve low effort demands or motivational components, and are less prone to expectancy-driven changes (Blokland, 2023; Rooney et al., 2024; Winkler & Hermann, 2019). Similarly, a weaker or less immersive placebo manipulation, such as the procedure used here, might not be sufficient to elicit engagement, particularly among older adults tested remotely. To better understand how placebo effects can be elicited in older adults, future studies should explore whether reinforcing positive expectations through more robust induction methods—such as combining verbal suggestion with associative learning by providing participants with tangible “evidence” of cognitive benefits (Parong et al., 2022; Piedimonte et al., 2024)—can enhance the placebo effect, even in an online environment. Additionally, examining the role of individual differences in moderating expectancy effects may help clarify the conditions under which placebo effects can be reliably observed in older adults.

Some limitations concerning demographic disparities between groups should be acknowledged. In the auditory oddball task analyses, the groups differed significantly in both SATURN scores and gender composition, whereas in the visual oddball task, only gender differences reached significance. While SATURN scores may have influenced baseline performance in the auditory task—potentially explaining why the Nocebo group performed better at PRE—gender difference is unlikely to have significantly affected results. This imbalance was primarily between the Control and Placebo groups (e.g., a higher proportion of males in the Control group), which nonetheless showed similar outcomes overall. However, given the limited and uneven gender distribution, no subgroup analyses by gender could be conducted. Additionally, the study was not pre-registered, which limits the a priori specification of hypotheses and analytical decisions.

In conclusion, the findings highlight an asymmetry in expectancy effects among older adults, with verbal suggestions eliciting a nocebo effect on performance, but not a placebo response. This suggests that nocebo effects can arise in a perceptual/attentional task even in the absence of social interaction. Instead, placebo effects may require stronger validation to influence cognition and translate into measurable improvements, pointing to the speculation that placebo and nocebo effects emerge through a combination of distinct mechanisms. The absence of an objective placebo effect in our study does not necessarily imply that such an effect cannot emerge in an online setting or in older adults; rather, expectancy induction methods may need to be more robust to counteract the lack of face-to-face interaction as well as individual differences. Understanding the conditions under which expectancy effects emerge has important implications for clinical and behavioral interventions and aging research. Future studies should refine online verbal suggestions, explore the role of individual differences, and determine the cognitive domains most susceptible to these effects, ultimately shedding light on the mechanisms underlying expectancy-driven changes in cognition.

## Supplementary Information

Below is the link to the electronic supplementary material.

Supplementary Material 1(DOCX 4.84 MB)

## Data Availability

The datasets used and analyzed during the current study are available from the corresponding author upon request.

## Abbreviations

SATURN: Self-Administered Tasks Uncovering Risk of Neurodegeneration
AP: Anti-Phase
3AFC: three-Alternative Forced-Choice
HP: Huggins Pitch
LPD: Logical Pixel Density
SOA: Stimulus Onset Asynchrony
RGB: Red Green Blue
IQR: interquartile range
