# Supplementary material for: Beyond expectations: nocebo suggestion affects cognitive performance in older adults
Source: Psychol Res. 2026 May 15;90(3):92. doi: 10.1007/s00426-026-02296-4 (PMC13179200; doi:10.1007/s00426-026-02296-4)
Supplement: Supplementary file 1 — Supplementary Material 1(DOCX 4.84 MB) [file 426_2026_2296_MOESM1_ESM.docx]

**Supplementary materials for:**

**“Beyond Expectations: Nocebo Suggestion Affects Cognitive Performance in Older Adults”**

INDEX:

1. Verbal suggestion
2. Expectation and efficacy perception questions and response format
3. Demographic characteristics and screening variables analyses
4. Overall mean difficulty level for PRE and POST performances analyses
5. Maximum difficulty level for PRE and POST performances analysis
6. Preliminary analyses – Control, Nocebo, and Placebo groups
7. Preliminary analyses – Nocebo and Placebo believers
8. Additional analyses for expectation and efficacy perception
9. Verbal suggestion

- Placebo

You have been allocated to the treatment group. As a result, you will receive a specific sound frequency for several minutes before starting the second part of the experiment.

Precisely, you are going to receive a treatment consisting of 200hz sound stimulation. The effect of the sound treatment is to enhance your ability to concentrate and to increase your level of attention crucial for the oddball task. Previous studies on healthy participants, and also in patients with attention-deficit disorder, have shown an improvement in attention and concentration after receiving such sound stimulation.

These advantages are also associated with positive changes in the brain. Namely, brain activity can be potentiated by sound stimulation and, in turn, some cognitive functions, like attention and concentration, can be boosted. As an example, an electroencephalography (EEG) study showed that four minutes of 200hz sound stimulation increases the attentional network activity in the brain for up to 1 hour. Here, we want to evaluate the effectiveness of this well-known four-minute sound stimulation (200hz) in an online setting. By exploring the potential drawbacks associated with auditory stimulation, we can gain a more comprehensive understanding of the challenges people face in online environments. This knowledge is essential, as it equips us with insights into the nuances of cognitive functioning and helps us develop targeted interventions.

- Nocebo

You have been allocated to the treatment group. As a result, you will receive a specific sound frequency for several minutes before starting the second part of the experiment.

Precisely, you will receive a treatment consisting of 200hz sound stimulation to decrease your ability to concentrate and your level of attention. Previous studies on healthy participants and clinical studies, like obsessive disorder, have shown a decline in attention and concentration after receiving such sound stimulation.

These effects can also be demonstrated in the brain. The brain can adapt neural connections, allowing some sections of the brain to function with little capacity. Thus, cognitive abilities connected with those specific brain areas can also be decreased at the same time. As an example, an electroencephalography (EEG) study showed that four minutes of 200hz sound stimulation can successfully decrease the attentional network activity at the frontal region for up to 1 hour. Here, we want to evaluate the effectiveness of this well-known four-minute sound stimulation (200hz) in an online setting. By exploring the potential drawbacks associated with auditory stimulation, we can gain a more comprehensive understanding of the challenges people face in online environments. This knowledge is essential, as it equips us with insights into the nuances of cognitive functioning and helps us develop targeted interventions. In addition, we want to clarify that these negative effects are completely reversible in the short term.

- Control

You have been allocated to the control group. As a result, you will be asked to listen to an inert sound for several minutes before starting the second part of the experiment.

The control group plays a crucial role as it provides a reference point for comparison with other experimental groups. Due to this, we need to maintain the experiment and the physical properties of the auditory stimulus as similar as possible. In particular, the sound that you are going to listen to, which is a pure tone of 200hz, does not modulate any cognitive function, like attention or concentration required for the oddball task. In addition, this sound will be played for a brief period, just 4 minutes.

1. Expectation and efficacy perception questions and response format

- Expectation

Question:

“Before starting again with the visual and auditory tasks, we would like to ask you one more question regarding the 4-minute sound stimulation that you have just listened to. Do you expect your performance to change after listening to the sound?”

Response format:

- “Yes, I expect my performance to improve”
- “Yes, I expect my performance to get worse”
- “No, I do not expect my performance to change”
- Efficacy perception

Question:

“Did you notice any changes in your performance after listening to the 4-minute sound stimulation?”

Response format:

- “Yes, my performance got better”
- “Yes, my performance got worse”
- “No, my performance did not change”

1. Demographic characteristics and screening variables analysis

Unimodal condition

In the auditory oddball task, the one-way ANOVAs on key demographic and screening variables revealed no significant group differences in Age, *F*(2, 76) = .662, *p* = .519, η^2^_p_ = .017, or Years of Education, *F*(2, 76) = .579, *p* = .563, η^2^_p_ = .015. Similarly, the proportion of participants passing AP, HP, or combined pre-screening tests did not significantly differ between groups (all *p*s > .649).

However, a significant group difference was observed for gender distribution, *F*(2, 76) = 4.793, *p* = .011, η^2^_p_ = .112. Post hoc comparisons using Bonferroni correction revealed that the Control group had a higher proportion of female participants than the Placebo group, *t*(76) = 2.874, *p* = .016), with no significant differences between the other groups (*p*s > .051).

Group differences were also found for performance on the SATURN test, *F*(2, 76) = 3.304, *p* = .042, η^2^_p_ = .08. Post hoc analysis indicated that Control participants scored significantly lower than Nocebo participants, *t*(76) = -2.57, *p* = .036, while comparisons between Placebo and the other groups were not significant (*p*s > .527).

Crossmodal condition

In the visual oddball task, one-way ANOVAs on key demographic and screening variables revealed no significant group differences in Age, *F*(2, 83) = .23, *p* = .795, η^2^_p_ = .006, Years of Education, *F*(2, 83) = .511, *p* = .602, η^2^_p_ = .012, or SATURN scores, *F*(2, 83) = 2.261, *p* = .111, η^2^_p_ = .052. Similarly, the proportion of participants passing AP, HP, or combined pre-screening tests did not significantly differ between groups (all *p*s > .679).

However, a significant group difference emerged for gender distribution, *F*(2, 83) = 7.342, *p* = .001, η^2^_p_ = .15. Post hoc comparisons with Bonferroni correction revealed that the Control group had a significantly higher proportion of female participants than both the Nocebo, *t*(83) = 2.851, *p* = .017, and Placebo, *t*(83) = 3.654, *p* = .001, groups. No significant difference in Gender was observed between the Nocebo and Placebo groups, *t*(83) = .754, *p* = 1.

1. Overall mean difficulty level for PRE and POST performances analyses

Unimodal condition

In the auditory oddball task (see Figure S1), a one-way ANOVA on overall mean difficulty level at PRE with Group as between-subjects factor revealed no evidence that overall mean difficulty levels significantly differed between the groups, *F*(2, 76) = 2.172, *p* = .121, η^2^ = .054. Therefore, no delta (POST – PRE) of overall mean difficulty level was computed.

The marginal ANOVA of the linear mixed-effects model examining overall mean difficulty level, with Group as a between-subjects factor, Session as a within-subject factor, and Participant as a random effect, revealed no significant main effect of Group, *F*(2, 100.51) = 2.205, *p* = .116, η^2^_p_ = .042, or Session, *F*(1, 76) = .008, *p* = .927, η^2^_p_ < .001. The Group × Session interaction was also non-significant, *F*(2, 76) = .413, *p* = .663, η^2^_p_ = .011, indicating that the overall mean difficulty level did not vary as a function of session or group, nor did it differ in its change across sessions between groups.

**Figure S1**

*Auditory oddball task – Overall mean difficulty level at PRE- and POST-intervention.*


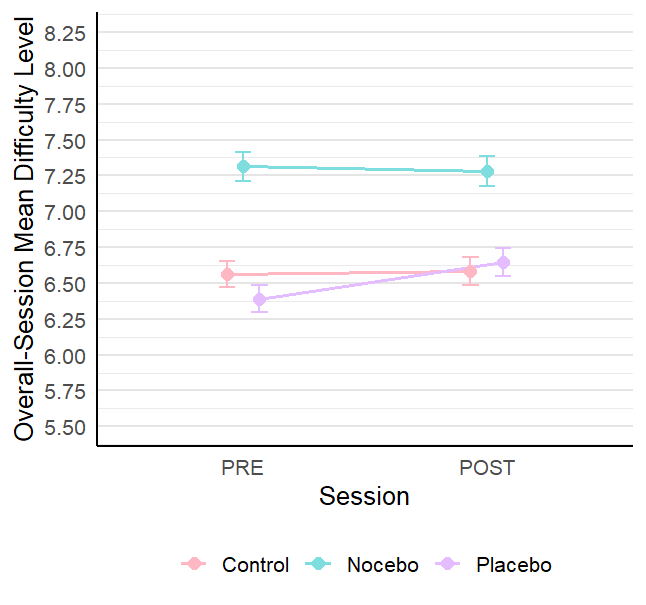


*Note.* The y-axis presents a limited portion of the full difficulty levels scale.

Crossmodal condition

In the visual oddball task (see Figure S2), a one-way ANOVA on overall mean difficulty level at PRE with Group as between-subjects factor revealed no evidence that overall mean difficulty levels significantly differed between the groups, *F*(2, 83) =.023, *p* = .977, η^2^ = .001. Therefore, no delta (POST – PRE) of overall mean difficulty level was computed.

The marginal ANOVA of the linear mixed-effects model examining overall mean difficulty level, with Group as a between-subjects factor, Session as a within-subject factor, and Participant as a random effect, revealed no significant main effect of Group, *F*(2, 134.95) =.015, *p* = .985, η^2^_p_ = .02, or Session, *F*(1, 83) = 2.77, *p* = .1, η^2^_p_ < .001. The Group × Session interaction was only marginally significant, *F*(2, 83) = 2.517, *p* = .087, η^2^_p_ = .06, indicating that the overall mean difficulty level did not vary as a function of session or group, nor did it differ in its change across sessions between groups.

**Figure S2**

*Visual oddball task – Overall mean difficulty level at PRE- and POST-intervention.*


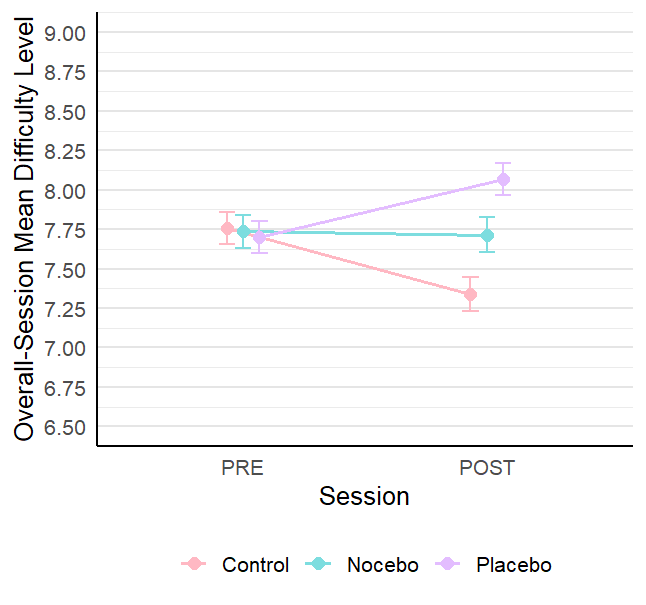


*Note.* The y-axis presents a limited portion of the full difficulty levels scale.

1. Maximum difficulty level for PRE and POST performances analyses

Unimodal condition

In the auditory oddball task (see Figure S3), a one-way ANOVA on maximum difficulty level at PRE with Group as between-subjects factor revealed no evidence that maximum difficulty levels differed significantly between groups, *F*(2, 76) = 1.629, *p* = .203, η^2^ = .041. Therefore, no delta (POST – PRE) of overall mean difficulty level was computed.

The marginal ANOVA of the linear mixed-effects model examining the maximum difficulty level, with Group as a between-subjects factor, Session as a within-subject factor, and Participant as a random effect, revealed no significant main effect of Group, *F*(2, 98.326) = 1.513, *p* = .225, η^2^_p_ = .03, or Session, *F*(1, 76) = .823, *p* = .367, η^2^_p_ = .01. The Group × Session interaction was also non-significant, *F*(2, 76) = .226, *p* = .798, η^2^_p_ = .006, indicating that the maximum difficulty level did not vary as a function of session or group, nor did it differ in its change across sessions between groups.

**Figure S3**

*Auditory oddball task – Maximum difficulty level at PRE- and POST-intervention.*


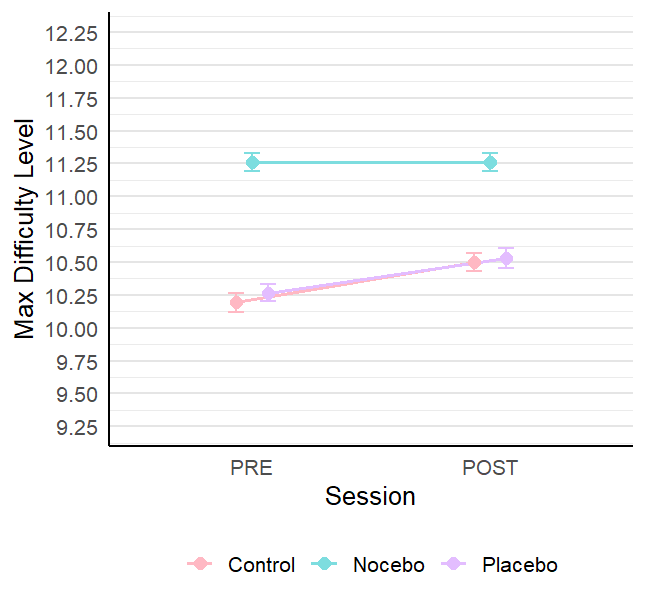


*Note.* The y-axis presents a limited portion of the full difficulty levels scale.

Crossmodal condition

In the visual oddball task (see Figure S4), a one-way ANOVA on maximum difficulty level at PRE with Group as between-subjects factor revealed no evidence that maximum difficulty levels significantly differed between the groups, *F*(2, 83) = .391, *p* = .678, η^2^ = .009. Therefore, no delta (POST – PRE) of overall mean difficulty level was computed.

The marginal ANOVA of the linear mixed-effects model examining the maximum difficulty level, with Group as a between-subjects factor, Session as a within-subject factor, and Participant as a random effect, revealed no significant main effect of Group, *F*(2, 125.77) = .302, *p* = .74, η^2^_p_ = .03, or Session, *F*(1, 83) = 1.678, *p* = .199, η^2^_p_ = .002. The Group × Session interaction was also non-significant, *F*(2, 83) = 1.739, *p* = .182, η^2^_p_ = .04, indicating that the maximum difficulty level did not vary as a function of session or group, nor did it differ in its change across sessions between groups.

**Figure S4**

*Visual oddball task – Maximum difficulty level at PRE- and POST-intervention.*


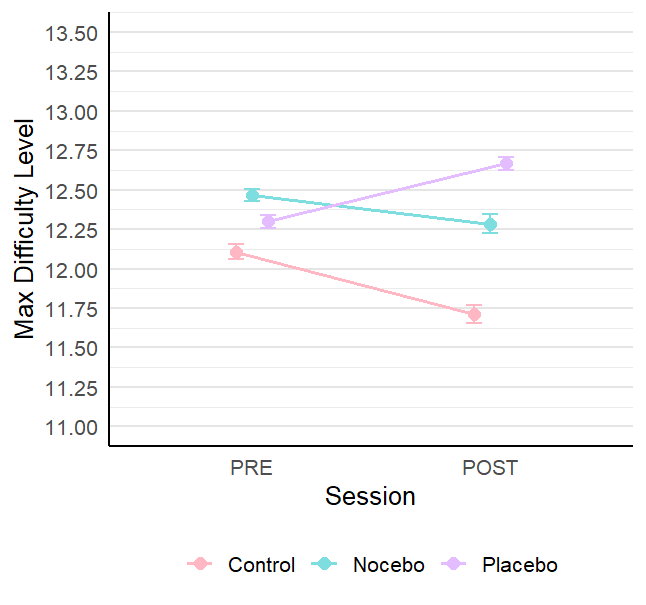


*Note.* The y-axis presents a limited portion of the full difficulty levels scale.

1. Preliminary analyses – Control, Nocebo, and Placebo groups

Unimodal condition

In the auditory oddball task (see Figure S5), preliminary analyses to evaluate the effect of trial progression on performance at baseline (PRE) across the three groups revealed a significant main effect of Trial, 𝐹(1, 3315) = 1587.864, *p* < .001, η^2^_p_ = .634, indicating that difficulty levels changed significantly across trials over time suggesting learning, as expected. There was no significant main effect of Group, 𝐹(2, 90.6) = .239, *p* = .788, η^2^_p_ = .005, but because of a significant interaction between Trial and Group, 𝐹(2, 3315) = 30.187, *p* < .001, η^2^_p_ = .018, post-hoc pairwise comparisons between groups for trial trends were conducted, revealing statistically significant differences after Bonferroni adjustment for multiple comparisons when contrasting the Control and the Nocebo groups, *t*(3315) = -7.131, *p* < .001, and the Nocebo and the Placebo groups, *t*(3315) = 6.489, *p* < .001, but not the Control and the Placebo groups, *t*(3315) = - .906, *p* = 1.

**Figure S5**

*Auditory oddball task – Mean difficulty level across trials at PRE.*


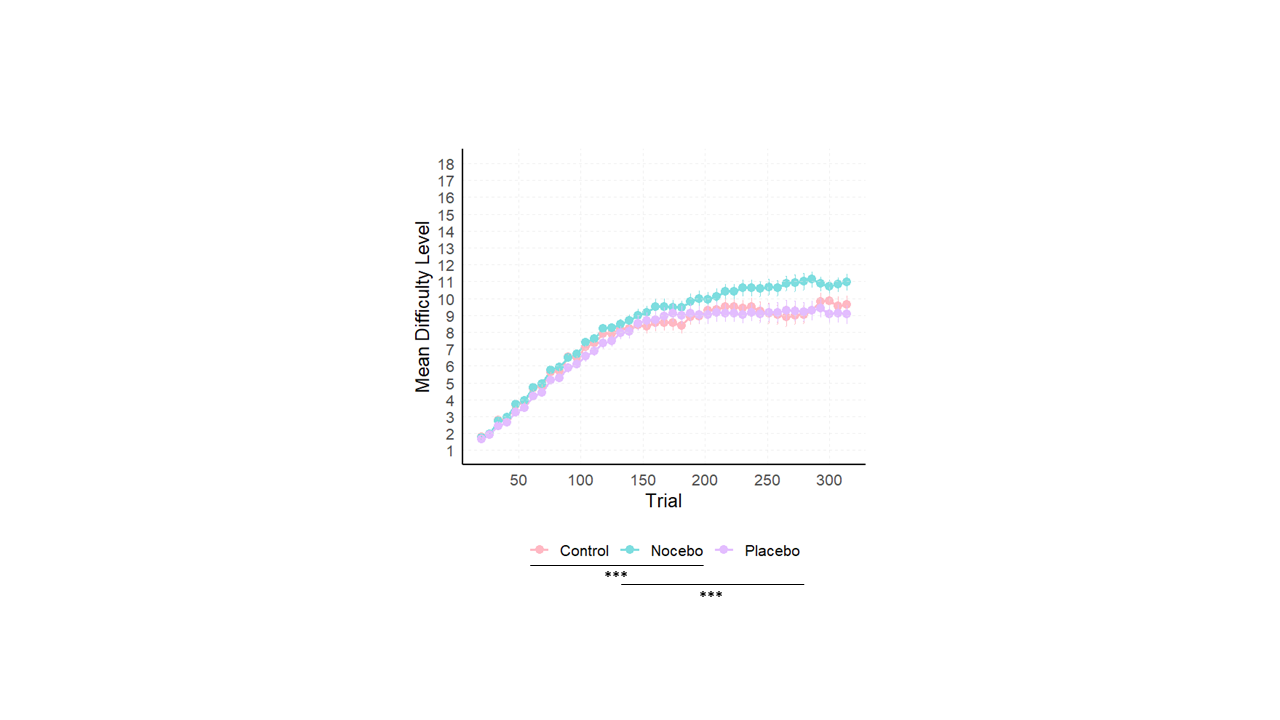


*Note*. Significance codes: ‘***’ for *p* < .001

Crossmodal condition

In the visual oddball task (see Figure S6), preliminary analyses to evaluate the effect of trial progression on performance at baseline (PRE) across the three groups revealed a significant main effect of Trial, 𝐹(1, 3609) = 3798.814, *p* < .001, η^2^_p_ = .781, indicating that difficulty levels changed significantly across trials over time suggesting learning, as expected. There was no significant main effect of Group, 𝐹(2, 119) = 1.535, *p* = .22, η^2^_p_ = .025, but because of a significant interaction between Trial and Group, 𝐹(2, 3609) = 7.785, *p* < .001, η^2^_p_ = .004, post-hoc pairwise comparisons between groups for trial trends were conducted, revealing statistically significant differences after Bonferroni adjustment for multiple comparisons when contrasting the Control and the Nocebo groups, *t*(3609) = -3.612, *p* = .001, and the Control and the Placebo groups, *t*(3609) = -3.205, *p* = .004, but not the Nocebo and the Placebo groups, *t*(3609) = .469, *p* = 1.

**Figure S6**

*Visual oddball task – Mean difficulty level across trials at PRE.*


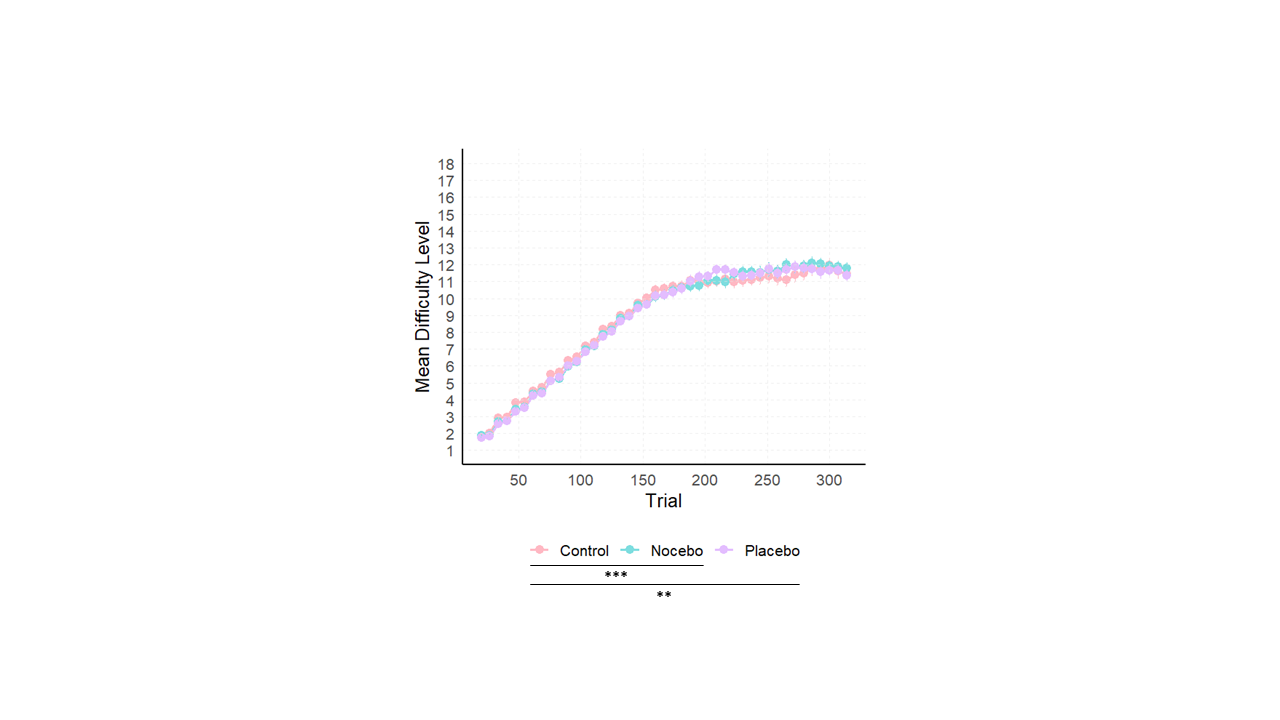


*Note*. Significance codes: ‘***’ for *p* < .001, ‘**’ for *p* < .01

1. Preliminary analyses – Nocebo and Placebo believers

Unimodal condition

In the auditory oddball task (see Figure S7), preliminary analyses to evaluate the effect of trial progression on performance at baseline (PRE) between the two believer groups revealed a significant main effect of Trial, 𝐹(1, 796) = 1670.925, *p* < .001, η^2^_p_ = .821, indicating that difficulty levels changed significantly across trials over time suggesting learning, as expected. There was no significant main effect of Group, 𝐹(1, 24.48) = .704, *p* = .41, η^2^_p_ = .028, but a significant interaction between Trial and Group, 𝐹(1, 796) = 20.894, *p* < .001, η^2^_p_ = .026.

**Figure S7**

*Auditory oddball task – Mean difficulty level across trials across the two believer groups.*


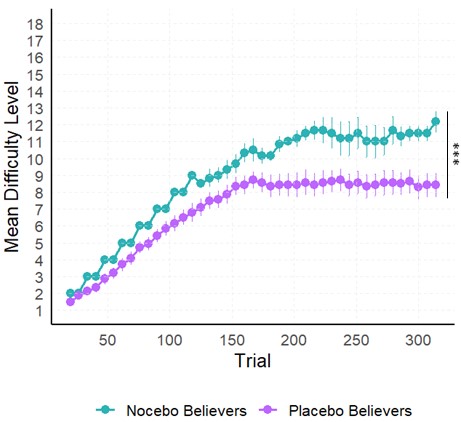


*Note*. Significance code: ‘***’ for *p* < .001

Crossmodal condition

In the visual oddball task (see Figure S8), preliminary analyses to evaluate the effect of trial progression on performance at baseline (PRE) between the two believer groups revealed a significant main effect of Trial, 𝐹(1, 838) = 753.663, *p* < .001, η^2^_p_ = .647, indicating that difficulty levels changed significantly across trials over time suggesting learning, as expected. There was no significant main effect of Group, 𝐹(1, 21.62) = .281, *p* = .601, η^2^_p_ = .013, but a significant interaction between Trial and Group, 𝐹(1, 838) = 45.745, *p* < .001, η^2^_p_ = .052.

**Figure S8**

*Visual oddball task – Mean difficulty level across trials across the two believer groups.*


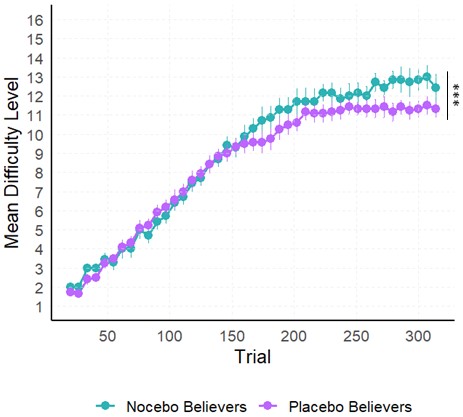


*Note*. Significance code: ‘***’ for *p* < .001

1. Additional analyses for expectation and efficacy perception

To ensure that the observed pattern of subjective responses was not dependent on the categorical treatment of expectancy and perceived efficacy, we conducted additional analyses treating magnitude ratings as numerical scores on a Likert scale. Furthermore, given that expectancy and perceived efficacy were assessed with a single set of items referring to the intervention in general, we performed complementary analyses aggregating responses across task modalities. These analyses serve as robustness checks of the main findings.

- Numerical scoring of expectancy and perceived efficacy

Expectancy and perceived efficacy were assessed using a two-step procedure. First, participants indicated the direction of their expectancy/perceived efficacy (improvement, worsening, or no change, as already written in section 2 of this file). If a change was indicated, participants rated its magnitude on a five-point Likert scale (from slightly to extremely).

To examine whether results depended on the directional treatment of responses, magnitude ratings were transformed into a discrete scale ranging from −5 (indicating expecting extremely worse performance or having perceived extremely worse performance) to +5 (indicating expecting extremely improved performance or having perceived extremely improved performance), with 0 representing no expected change. Separate one-way ANOVAs were conducted for each condition and outcome, followed by Bonferroni-corrected post-hoc comparisons (estimated marginal means).

Unimodal condition

The ANOVA on expectancy ratings revealed a significant main effect of Group, *F*(2, 76) = 9.442, *p* < .001. Post-hoc comparisons indicated significant differences between the Control and Placebo groups, *t*(76) = −3.48, *p* = .003, and between the Nocebo and Placebo groups, *t*(76) = −3.888, *p* = .001. No significant difference emerged between the Control and Nocebo groups, *t*(76) = 0.507, *p* = 1

**Figure S9**

*Auditory oddball task – Distribution of expectancy ratings by group.*


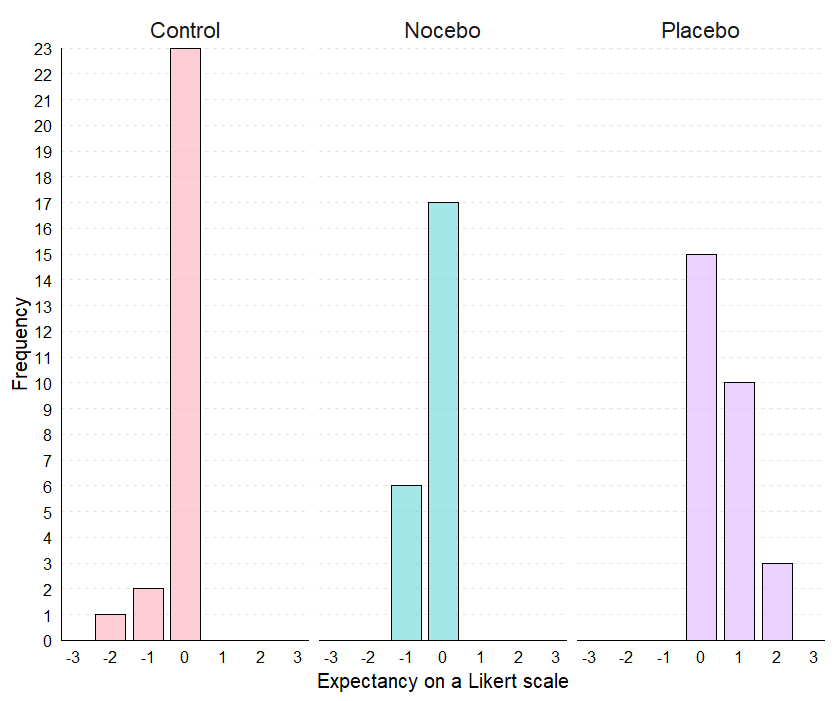


*Note*. Although responses were coded on a −5 (indicating expecting extremely worse performance) to +5 (indicating expecting extremely improved performance) scale, the displayed range was truncated to −3 to +3 given the absence of observations at the extreme scale points across groups.

Regarding perceived efficacy ratings, a significant main effect of Group was also observed, *F*(2, 76) = 5.853, *p* = .004. Post-hoc comparisons revealed a significant difference between the Control and Placebo groups, *t*(76) = −3.387, *p* = .003. No significant differences were found between the Control and Nocebo groups, *t*(76) = −1.28, *p* = .613, nor between the Nocebo and Placebo groups, *t*(76) = −1.953, *p* = .164.

**Figure S10**

*Auditory oddball task – Distribution of perceived efficacy ratings by group.*


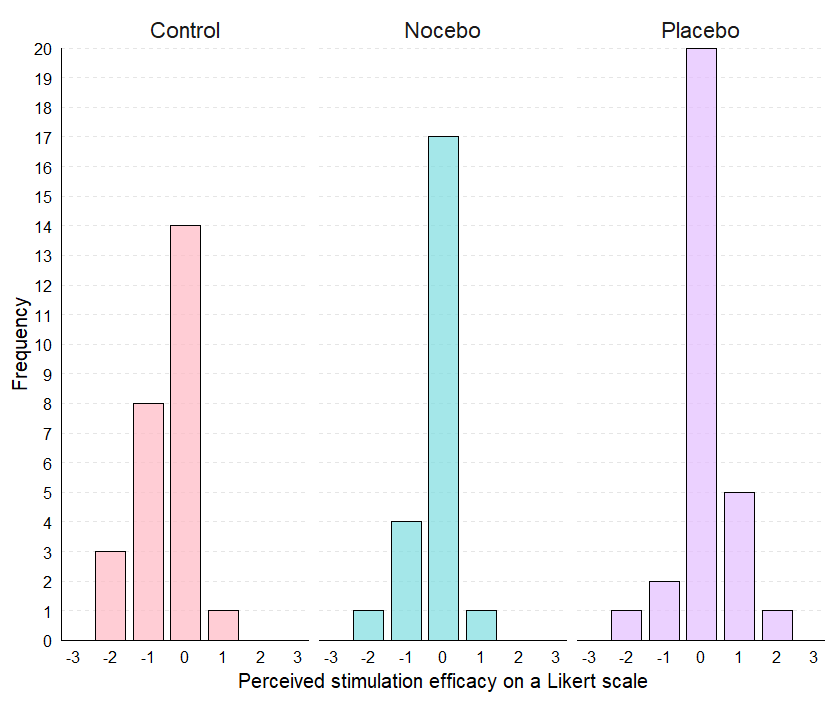


*Note*. Although responses were coded on a −5 (indicating having perceived extremely worse performance) to +5 (indicating having perceived extremely improved performance) scale, the displayed range was truncated to −3 to +3 given the absence of observations at the extreme scale points across groups.

Crossmodal condition

The ANOVA on expectancy ratings revealed a significant main effect of Group, *F*(2, 83) = 7.328, *p* = .002. Post-hoc comparisons showed significant differences between the Control and Placebo groups, *t*(83) = −2.495, *p* = .044, and between the Nocebo and Placebo groups, *t*(83) = −3.747, *p* = .001. The comparison between the Control and Nocebo groups was not significant, *t*(83) = 1.232, *p* = .665.

**Figure S11**

*Visual oddball task – Distribution of expectancy ratings by group.*


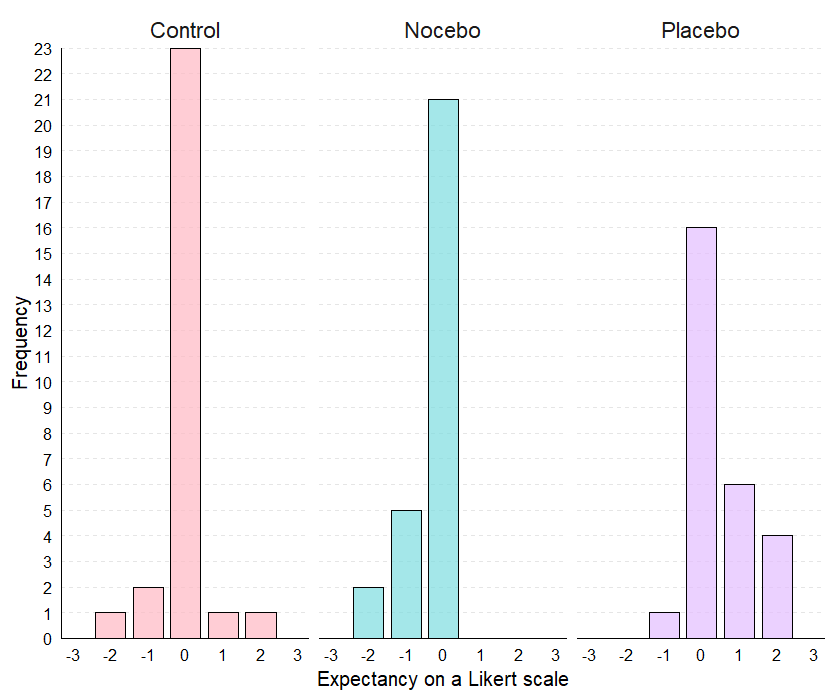


*Note*. Although responses were coded on a −5 (indicating expecting extremely worse performance) to +5 (indicating expecting extremely improved performance) scale, the displayed range was truncated to −3 to +3 given the absence of observations at the extreme scale points across groups.

For perceived efficacy ratings, a significant main effect of Group emerged, *F*(2, 83) = 4.202, *p* = .018. Post-hoc tests revealed a significant difference between the Control and Placebo groups, *t*(83) = −2.878, *p* = .015. Comparisons between the Control and Nocebo groups, *t*(83) = −1.167, *p* = .740, and between the Nocebo and Placebo groups, *t*(83) = −1.691, *p* = .284, were not significant.

**Figure S12**

*Visual oddball task – Distribution of perceived efficacy ratings by group.*


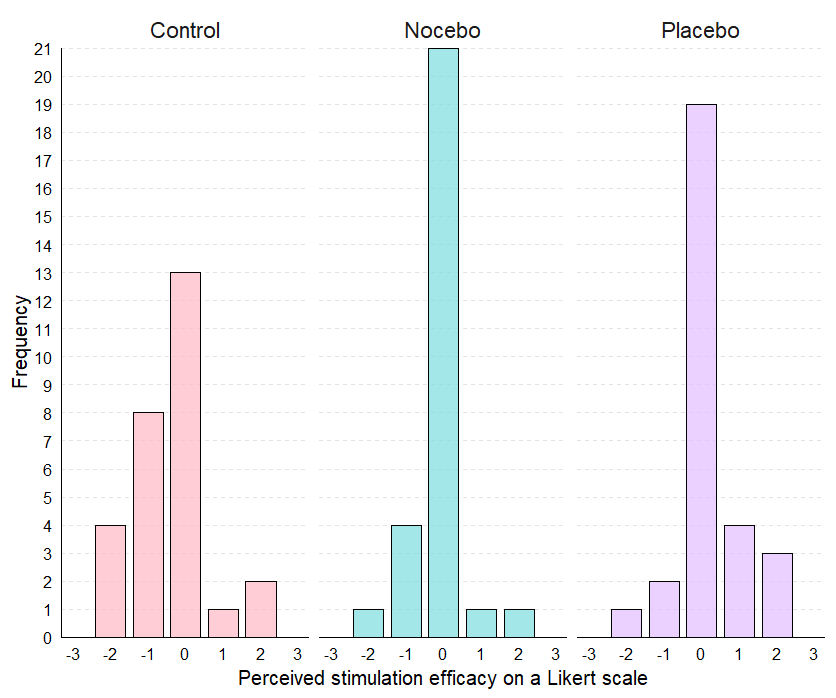


*Note*. Although responses were coded on a −5 (indicating having perceived extremely worse performance) to +5 (indicating having perceived extremely improved performance) scale, the displayed range was truncated to −3 to +3 given the absence of observations at the extreme scale points across groups.

Overall, the pattern of results mirrors those reported in the main manuscript, indicating that findings are robust to the treatment of expectancy and perceived efficacy as categorical variables or as numerical scores on the -5 to + scale.

- Aggregated expectancy and perceived efficacy across modalities

Because expectancy and perceived efficacy were assessed with a single set of questions referring to the intervention in general (rather than modality-specific tasks), we conducted complementary analyses combining participants across unimodal and crossmodal conditions.

A Fisher’s Exact Test revealed a significant association between Group and reported expectancy direction, *p* < .001 (see Table S1 for the absolute and relative frequencies of expectancy endorsements across groups). Post-hoc pairwise comparisons revealed a significant difference between the Control and Placebo groups when comparing “Expectancy of improvement” vs. “Expectancy of no change”, *p* = .002; no significant difference emerged between “Expectancy of worsening” and “Expectancy of no change”, *p* = 1, or “Expectancy of improvement” vs. “Expectancy of worsening”, *p* = .128. Although approximately half of the participants in the Placebo group still reported expecting no change, a notably larger proportion (48.6%) reported expecting improvement compared to the Control group (6.9%), suggesting that Placebo participants were substantially more inclined to anticipate an improvement in their performance than those in the Control group. This pattern indicates that the verbal suggestion was effective in increasing improvement-related expectancies among Placebo participants.

The comparison between the Control and Nocebo groups revealed no significant differences in any expectancy level, with all *p*s > .38.

**Table S1**

*Absolute frequencies of expectancy endorsements by participants in each group, irrespective of condition (unimodal or crossmodal)*

|  | Expectancy of worsening | No change expected | Expectancy of improvement | **Total** |
| --- | --- | --- | --- | --- |
| Control | 3 (10.3%) | 24 (82.8%) | 2 (6.9%) | 29 |
| Nocebo | 8 (26.7%) | 22 (73.3%) | 0 | 30 |
| Placebo | 2 (5.7%) | 16 (45.7%) | 17 (48.6%) | 35 |
| **Total** | 13 | 62 | 19 | 94 |

*Note*. Relative frequencies are shown in parentheses and represent within-group proportions (i.e., calculated as percentages of the total number of participants in each group).

The results of the Fisher’s Exact Test indicated a significant association between group assignment and perceived efficacy of the intervention, *p* = .005 (see Table S2 for the absolute and relative frequencies of perceived efficacy endorsements across groups). Post-hoc pairwise comparisons revealed a significant difference between the Control and Placebo groups specifically in the distribution of “Perception of improvement” versus “Perception of worsening”, *p* = .02, and “Perception of worsening” versus “Perception of no change”, *p* = .038. While approximately half of the Control group and the majority of the Placebo group reported to have not perceived a change in their performance, fewer participants in the Placebo group reported a perceived worsening (8.5%) compared to the Control group (41.4%). Conversely, a greater proportion of Placebo participants (28.6%) reported perceiving an improvement in their performance compared to only 10.3% in the Control group. These patterns suggest that Placebo participants were more inclined to perceive an improvement and less likely to report a decline in performance.

The comparison between the Control and Nocebo groups revealed no significant differences in any level of perceived efficacy, all *p*s > .05.

**Table S2**

*Absolute frequencies of perceived efficacy endorsements by participants in each group, irrespective of condition (unimodal or crossmodal)*

|  | Perception of worsening | Perception of no change | Perception of improvement | **Total** |
| --- | --- | --- | --- | --- |
| Control | 12 (41.4%) | 14 (48.3%) | 3 (10.3%) | 29 |
| Nocebo | 5 (16.7%) | 23 (76.7%) | 2 (6.6%) | 30 |
| Placebo | 3 (8.5%) | 22 (62.9%) | 10 (28.6%) | 35 |
| **Total** | 20 | 59 | 15 | 94 |

*Note*. Relative frequencies are shown in parentheses and represent within-group proportions (i.e., calculated as percentages of the total number of participants in each group).

This aggregated analysis is consistent with the modality-specific findings reported in the main text and further supports the interpretation that expectancy modulation was primarily driven by the Placebo group.
